# Ultrathin, Soft, Bioresorbable Organic Electrochemical Transistors for Transient Spatiotemporal Mapping of Brain Activity

**DOI:** 10.1002/advs.202300504

**Published:** 2023-02-24

**Authors:** Mengge Wu, Kuanming Yao, Ningge Huang, Hu Li, Jingkun Zhou, Rui Shi, Jiyu Li, Xingcan Huang, Jian Li, Huiling Jia, Zhan Gao, Tsz Hung Wong, Dengfeng Li, Sihui Hou, Yiming Liu, Shiming Zhang, Enming Song, Junsheng Yu, Xinge Yu

**Affiliations:** ^1^ State Key Laboratory of Electronic Thin Films and Integrated Devices School of Optoelectronic Science and Engineering University of Electronic Science and Technology of China (UESTC) Chengdu 610054 P. R. China; ^2^ Department of Biomedical Engineering City University of Hong Kong Hong Kong P. R. China; ^3^ Shanghai Frontiers Science Research Base of Intelligent Optoelectronics and Perception Institute of Optoelectronics Fudan University Shanghai 200433 P. R. China; ^4^ Hong Kong Center for Cerebra‐Cardiovascular Health Engineering Hong Kong Science Park New Territories Hong Kong P. R. China; ^5^ Department of Electrical and Electronic Engineering The University of Hong Kong Hong Kong SAR P. R. China

**Keywords:** bioresorbable materials, multichannel electrophysiology mapping, neural interfaces, organic electrochemical transistor, transient electronics

## Abstract

A critical challenge lies in the development of the next‐generation neural interface, in mechanically tissue‐compatible fashion, that offer accurate, transient recording electrophysiological (EP) information and autonomous degradation after stable operation. Here, an ultrathin, lightweight, soft and multichannel neural interface is presented based on organic‐electrochemical‐transistor‐(OECT)‐based network, with capabilities of continuous high‐fidelity mapping of neural signals and biosafety active degrading after performing functions. Such platform yields a high spatiotemporal resolution of 1.42 ms and 20 µm, with signal‐to‐noise ratio up to ≈37 dB. The implantable OECT arrays can well establish stable functional neural interfaces, designed as fully biodegradable electronic platforms in vivo. Demonstrated applications of such OECT implants include real‐time monitoring of electrical activities from the cortical surface of rats under various conditions (e.g., narcosis, epileptic seizure, and electric stimuli) and electrocorticography mapping from 100 channels. This technology offers general applicability in neural interfaces, with great potential utility in treatment/diagnosis of neurological disorders.

## Graphical abstract

The ultrasoft, multichannel, biodegradable OECT‐based neural interfaces are developed, which not only monitor the μ‐ECoG signals in high fidelity, but also autonomous degradation without any trigger events. Arrays with 100 channels, temporal/spatial resolution of 1.42 ms and 20 µm, and great signal‐to‐noise ratio of 37 dB, are achieved, demonstrating a facile and efficient tool for diagnostic purposes and neuroscience research.

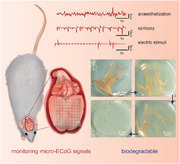

## 1 Introduction

Monitoring of electrophysiological (EP) signals of brain activities, especially during and 48 h after surgery, are critically important to guide surgery operation and reduce perioperative deaths.^[^ ^1^ ^]^ For instance, it can guide the administration of anesthetics, and avoid inadequate anesthesia and possible intraoperative traumatic awareness, allowing for precise patient‐centered anesthesia.^[^ ^2^ ^]^ Furthermore, postoperative brain activities monitoring is essential for predicting and assessing neurological complications after cardiovascular surgery.^[^ ^3^ ^]^ Delirium, the most crucial type of neurological complication after cardiovascular surgery, not only increases the number of perioperative adverse events but is also closely associated with long‐term cognitive impairment. The first 48 h after surgery was a high incidence of delirium, accounting for 87.2% of the total delirium incidence. From the third postoperative day onwards, the percentage of delirium incidence decreased rapidly.^[^ ^4^ ^]^ Recent neurophysiological studies have shown that intraoperative and postoperative EP signals changes are closely related to delirium in patients, manifesting as burst suppression and decreased alpha power.^[^ ^5^ ^]^ EP signals’ amplitude reduces, and frequency slows down when the mean cerebral blood flow is below 22 mL/100 g min⁻^1^. The changes in EP signals preceded the clinical diagnosis by 7 h and the CT examination by 44 hours, implying its excellent application prospects in perioperative prediction, assessment of patient physiological levels, and guidance of surgical operations.^[^ ^6^ ^]^

Neural interfaces that record EP signals rely on electrical mechanism and can be established by passive microelectrodes and active transistors.^[^ ^7^, ^8^, ^9^, ^10^ ^]^ Brain activities are of low amplitude and interfered with various ex/internal sources.^[^ ^11^ ^]^ To capture EP signal cleanly, strategy of optimizing materials selection and device design were introduced to reduce the impedance in passive microelectrodes.^[^ ^8^, ^12^ ^]^ Dion Khodagholy et al. proposed 16 × 16 neural interface where PEDOT: PSS/Au establishes an efficient abiotic/biotic interface, resulting in a signal‐to‐noise ratio (SNR) as high as 40 dB.^[^ ^13^ ^]^ Chia‐Han Chiang et al. developed an field‐effect transistors (FET)‐based neural matrix, where silicon dioxide served as the biofluid barrier and dielectric medium for capacitive coupling between the brain and electrode contacts.^[^ ^14^ ^]^ A 36 × 28 array was implanted in monkeys with an SNR of 2.4 dB. Besides, the emerging organic electrochemical transistors (OECT) constitute the promising candidate for next‐generation neural interfaces due to the unique advantages^[^ ^15^, ^16^ ^]^: (i) High transconductance (g_m_) values, on the order of milliSiemens (mS) for micrometer‐scale devices, at least two orders of magnitude higher than that of FET.^[^ ^17^ ^]^ Detailed explanation displays in Figure S1 (Supporting Information) and relevant Note. (ii) OECT is more flexible and ultrathin than FET, thanks to the lower Young's modulus of polymer semiconductors used. Such property allows OECT to establish a stable electrical and mechanical contact and minimize damage to biological tissues. (iii) OECT operates in low operation voltage (< 0.5 V), while it may need several even tens volts for FET and is available for low‐power bioelectronics.^[^ ^18^, ^19^ ^]^ In study of George G. Malliaras et al., OECT, PEDOT: PSS surface electrode, and Ir‐penetrating electrode were utilized to record the pathological activities.^[^ ^20^ ^]^ It turned out that OECT not only recorded the same information as passive electrodes, but also has a greater signal‐to‐noise ratio of 52.7 dB. In this context, the ultrathin, soft OECT can seamlessly adhere onto the cerebral cortex for continuous micro‐ electrocorticogram (μ‐ECoG) signals monitoring, with the reduced interface impedance and limited motion artifacts.

Two critical challenges in biodegradability and multichannel need to be addressed for OECT‐based neural interfaces that record brain activities in perioperative. Clinical requirements of biodegradable electronics’ operational lifetime range from a few days to weeks.^[^ ^21^ ^]^ The biodegradability erases the electronics without a trace, avoiding the risks, complications, and costs associated with the secondary removal surgery. The key to such bioelectronics is biodegradable materials, including dielectrics, conductors, and semiconductors.^[^ ^22^ ^]^ Functional materials ranging from polymers (such as polyhydroxyalkanoates,^[^ ^23^ ^]^ polylactic acid,^[^ ^24^ ^]^ and poly (butylene succinate)^[^ ^25^ ^]^), to metals^[^ ^26^, ^27^ ^]^ (such as Mg, Mo, and W), to inorganic materials^[^ ^28^, ^29^ ^]^(such as highly doped silicon and SiO_2_), providing strong support for biodegradable neural interfaces. Another huge challenge for OECT‐based neural interface is the data sample size, i.e., high‐density and multichannel array. It is well known that the structure of living organisms is sophisticated and complex, especially the brain, which is anatomically and functionally organized into separated regions. Therefore, elaborate designs of OECT‐based neural interfaces are necessary to realize a higher spatial resolution and molecular level recording so that doctors can obtain more accurate information and design personalized treatment for patients. The μ‐ECoG recordings with hundreds of and thousands of channels have been well‐established in previous studies.^[^ ^30^, ^31^ ^]^ By far, 16‐channel array is the most reported OECT array, which has been used to record signals from primary cardiomyocytes,^[^ ^32^ ^]^ ECG on the heart surface of rats,^[^ ^33^ ^]^ and acellular action potential.^[^ ^34^ ^]^ However, compared with hundreds and thousands of channels in microelectrode and FET, there is still massive room for improvement.

Here, we present an ultrathin, tissue‐compatible flexible, partly biodegradable neural interface with 100 channels for the short‐term monitoring the electrical activity from the rats’ cerebral cortex. This platform incorporates biodegradable materials, unique fabrication and integration strategies. Thus, the assembly of this neural interface on a millimeter scale enables cellular‐level mapping and the highest channels record for OECT array (Table S2, Supporting Information). We demonstrate that our transient OECT implants can: (i) Provide uniform and high transconductance up to 9.0 mS in vitro and record enhanced EP signals with SNR up to 37 dB in vivo; (ii) Perform 100 channels and harvest cellular‐level EP signals in a rat module; (iii) Identify the normal and pathological region of the animal model in active matrix‐addressed recording in vivo; (iv) Degrade spontaneously after completing its mission and avoid the secondary remove surgery.

## 2 Results

### 2.1 Structure of the Biodegradable OECT‐Based Neural Interface

**Figure 1:**
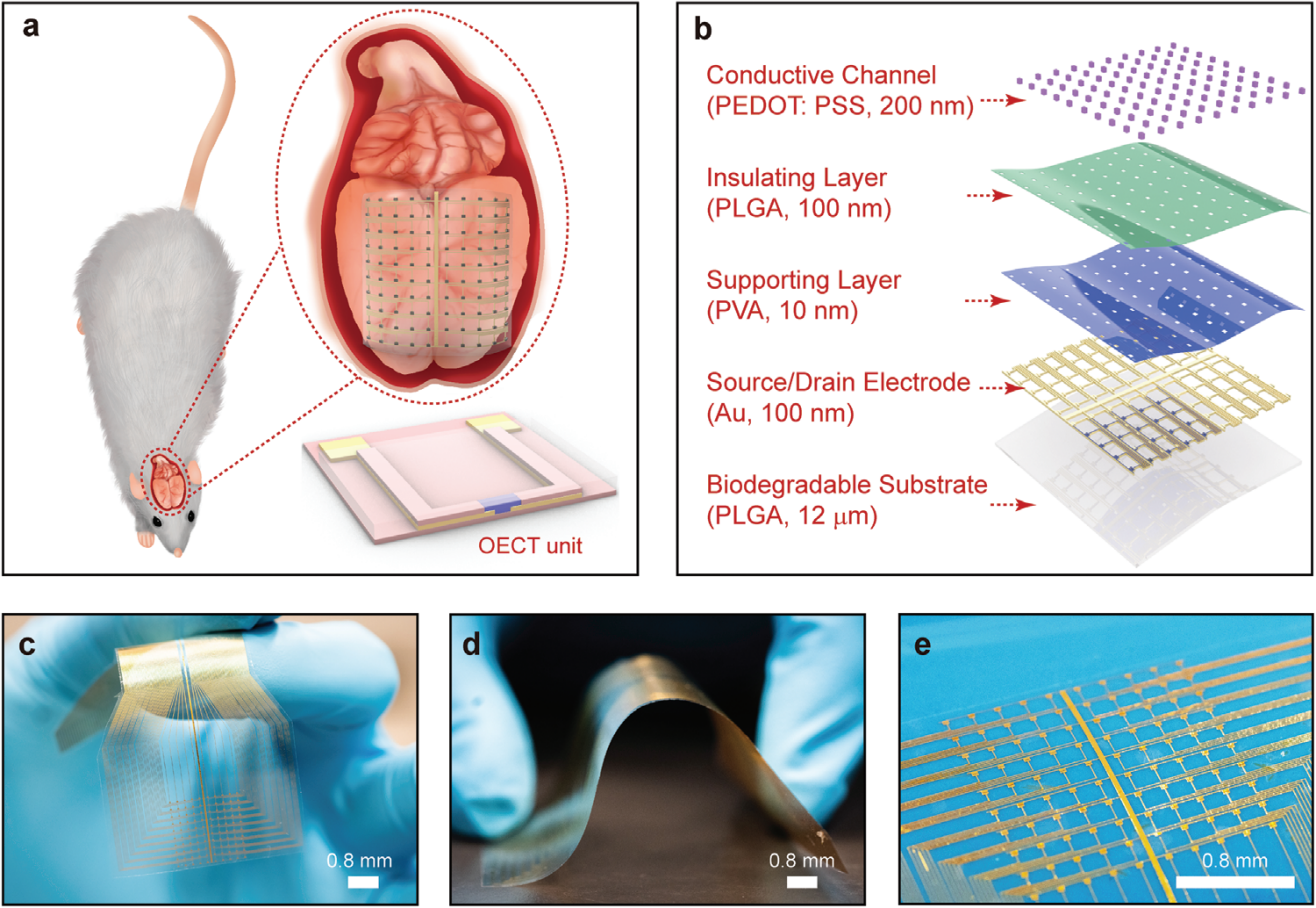
Ultrathin, soft, high‐throughput transient OECT Array as a core platform for a high‐fidelity brain‐machine interface. a) Cartoon illustrations of a transient OECT array placed on the cerebral cortex in the animal model for μ‐ECoG signals recording, and the OECT unit. b) Schematic explored‐view illustration of the transient OECT platform for high‐spatiotemporal resolution μ‐ECoG signals, in a fully biodegradable construction (thickness of ≈15 µm, and weight of 99.3 mg). This 8 × 8 mm^2^ active area includes 100 OECT units. c,d,e) Optical images to demonstrate the tissue‐compatible mechanical flexibility, ultrathin, and high‐density of biodegradable OECT array.

**Figure** **1**a provides an application scenario of this biodegradable OECT implant that softly laminates onto the rat's cerebral cortex to monitor the μ‐ECoG signals in real‐time with a single OECT unit inserted. Figure 1b illustrates the exploded schematic diagram of OECT array, which consists of biocompatible materials, including poly(lactic‐co‐glycolic acid) (PLGA),^[^ ^35^, ^36^ ^]^ Au electrode,^[^ ^37^ ^]^ and organic semiconductor of PEDOT: PSS.^[^ ^38^ ^]^ A water‐soluble PVA film (≈10 nm) deposited onto the source‐drain electrode to prevent PLGA insulting layer from dissolving the substrate. Dry‐etched defined PEDOT: PSS patterns as channel for electrochemical coupling neural signals, due to its excellent biocompatibility, electrochemical performance, and scalability properties (more details display in Figure S2 (Supporting Information) and Note). And detailed fabrication scheme appears in Methods and Figures S3–S6 (Supporting Information). This OECT array allows stable recording at 100 sites that arranged in 0.64 cm^2^, a general dimension of cerebral cortex of Sprague Dawley rats, with conductive channel size of 200 × 20 µm^2^ that is comparable to the neurons size from several to hundreds of microns.^[^ ^39^ ^]^ Low elastic modulus materials, ultrathin device architecture, and improved mechanical design determine the conformal contact against the curved surface of the cerebral cortex. Figure 1c,d demonstrates the ultra‐thin, lightweight, and flexible nature of the OECT implant. The minimum bending radius is less than 5 mm, and OECTs maintain stable performance under bend and unbent states (Figure S7, Supporting Information). Figure 1e presents a magnified image of active sensing region. All OECT units have independent drain electrodes, while share a common source electrode (grounded on the circuit) to reduce the size of the lineup.

### 2.2 In Vitro Characterization

**Figure 2:**
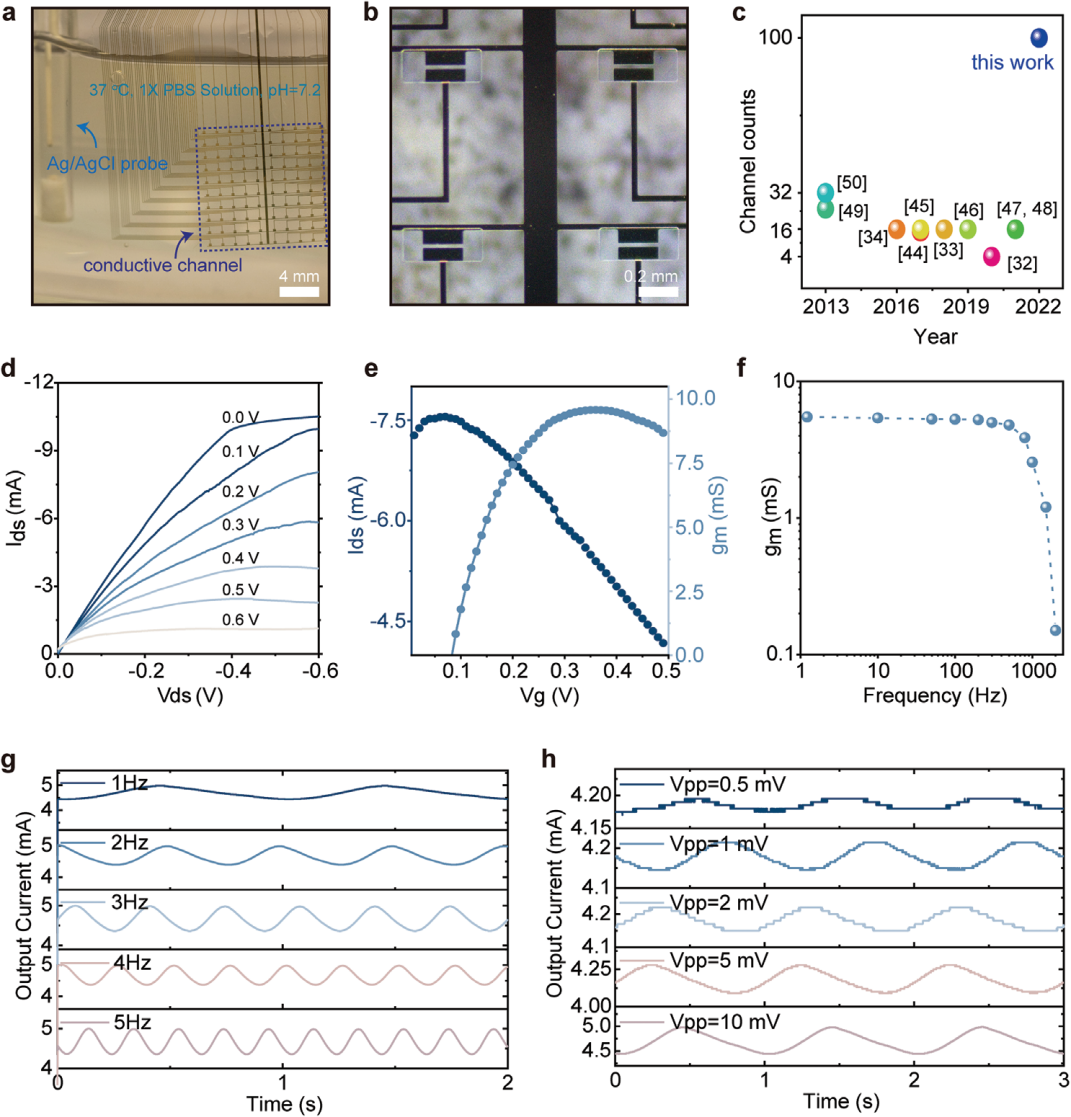
Characterization of the biodegradable transient OECT array in vitro. a) Optical image of measurement condition, where array immersing in 1X phosphate‐buffered saline (PBS) solution at 37 °C, pH = 7.2, and the Ag/AgCl probe serves as gate. b) Optical micrograph image of four‐unit cells at completed fabrication. c) Compare the channel count between this work and other OECT‐based biosensing applications in vivo.^[^ ^32^, ^33^, ^34^, ^44^, ^45^, ^46^, ^47^, ^48^, ^49^, ^50^ ^]^ d) Output, and e) Transfer characteristics for a representative PEDOT: PSS‐based OECT with conductive channel size of a 200 × 20 µm^2^. f) The relationship between transconductance and frequency, where the OECT was biased by applying ‐0.5 V at the drain terminal and 10 mV at the gate terminal, and a set of sine waves with frequency from 1 Hz to 2 KHz. g) Demonstrations of output signals in OECT unit related to input sine waves with amplitude of 10 mV at various frequencies. h) Demonstrations of output signals in OECT unit related to input sine waves with various amplitudes at 1 Hz.

The OECT array were characterized in vitro using 1X Phosphate Buffered Saline (PBS) solution (37 °C, pH = 7.2) as electrolyte, and Ag/AgCl probe as gate electrode (**Figure** **2**a and Figure S8, Supporting Information). The magnified optical microscope image of Figure 2b displays the high‐density OECT array in a more readable way. Figure 2c summaries the channel count of reported OECT array used in vivo, and it turns out that the 100 channels we proposed far exceeded the previous reports^[32‐34,44–50]^. More details are summarized in Table S2 (Supporting Information). The output characteristics of OECT unit is shown in Figure 2d, where typical plots of PEDOT: PSS‐based OECTs operated in the depletion regime can be observed. Upon trigger of a positive V_g_, cations in electrolyte enter the conductive channel and electrochemically doping it, increasing the impedance of PEDOT: PSS and decreasing the drain current (I_ds_). The transfer characteristics for V_ds_ = ‐0.5 V (Figure 2e) exhibit a downward trend as the V_g_ increases. The amplification capability of the OECT is evaluated by the transconductance value (g_m_). g_m_ is determined by the device geometry and the physicochemical properties of the channel materials, and it can be described as follows^[^ ^40^ ^]^:

(1) $$g_{m}=\frac{Wd}{L}\;\mu C^{*}\left(V_{\mathrm{th}}-V_{g}\right)$$

where W, d, and L are the channel width, thickness, and length, respectively. μ is the carrier mobility, C* is the capacitance per unit volume of the channel, and V_th_ is the threshold voltage. The maximum g_m_ value of ≈9.0 milliSiemens (mS), with a mean value of 8.67 mS and standard deviation of 0.30 mS, indicates a high uniformity of the array. Moreover, in Figure 2f, g_m_ values maintain at several mS when the sweep frequency is below 900 Hz that covers all possible μ‐ECoG activities (less than 500 Hz).^[^ ^41^ ^]^ These frequency responses are limited by the ion transport speed in ionic circuit and hole transport speed in electronic circuit, as shown in Figure S1 (Supporting Information).^[^ ^42^, ^43^ ^]^ In Figure 2g, the OECT unit can distinguish and record the input signals with different frequencies. Similarly, the responses to various peak‐to‐peak voltages (V_pp_) of input signals at 1 Hz are investigated, as displayed in Figure 2h. And the response time of OECT is ≈1.42 ms (Figure S9, Supporting Information). These results are comparable to the state‐of‐the‐art OECT technology based on PEDOT: PSS/Au (Table S3, Supporting Information). Therefore, the sensitive response to small V_g_ and a wide range of frequency, making it a superstar for monitoring μ‐ECoG signals that usually range from several hundred microvolts to a few millivolts.

**Figure 3:**
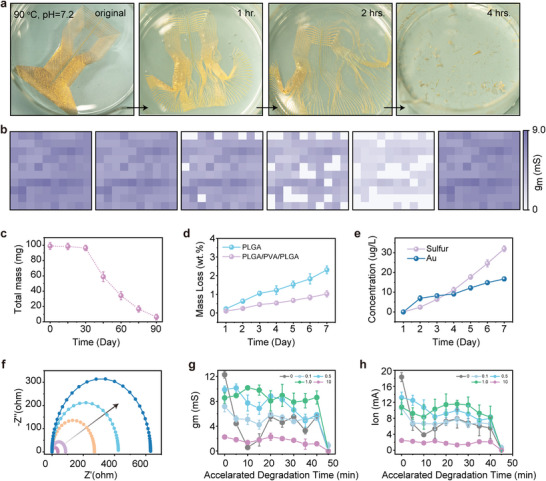
Characterization of the high‐throughput, transient OECT array mechanism. a) Photographs of devices collected at various times following immersion in 1X PBS (pH 7.2) solution at an elevated temperature (90 °C) for accelerated degradation. b) The change trend of the yield during the degradation process of the transient OECT array. c) Plot the relationship between total mass of the transient OECT platform with the time. d) Plot the mass loss relationship between pure PLGA (100 nm) and PLGA (12 µm)/PVA (10 nm)/PLGA (100 nm) with the time. e) Plot the relationship between concentration of sulfur element and Au in PBS with the immersion time. f) Electrochemical impedance spectrum of PEDOT: PSS versus time. (Data in (b)–(f) were collected during consecutive immersion in 1X PBS solution at 37 °C and pH = 7.2.). g) The relationship between the cross‐linked level of PEDOT: PSS and g_m_ at a gate voltage of 0.4 V. h) The relationship between the cross‐linked level of PEDOT: PSS and on‐state current (I_on_). (Data in (g)–(h) were collected during consecutive immersion in 1X PBS solution at 90 °C and pH = 7.2.)

Compared to natural sources,^[^ ^22^ ^]^ synthetic biodegradable polymers are more attractive options due to their controllable physical properties and degradation kinetics, as well as their evolutionary structural design and biological inert. PLGA decomposes into water and carbon dioxide by hydrolysis of its ester bonds in the presence of water, and rate is determined by temperature, pH value, and the ratio of lactic acid to glycolic acid.^[^ ^51^, ^52^ ^]^ **Figure** **3**a displays the results of accelerated degradation where OECT has been immersed in PBS solution at 90 °C. It took four hours to degrade the intact device into micro/nanoparticles that could be absorbed by or excreted from the human body. And Figure 3b illustrates the performance of degeneration kinetics for the OECT array soaked in 1X PBS at physiologically relevant temperature, evaluated by calculating g_m_ values at different times. In the first two days, the array yield was 100%, then decreased to 93%, 77%, 61%, and 36% in the following four days. The average g_m_ values decay from 8.9 mS on the first day to 4.0 mS on the sixth day. Typical Output and transfer curves captured every day are summarized in Figure S9 (Supporting Information). Figure 3c evaluates the degradation process of the OECT array's mass every 15 days. Observations indicate that the degradation process is non‐linear, slow in the first 30 days, and fast. After 90 days, only 10% of the mass remains. The reduction in device mass is determined by the degradation degree of PLGA. In detail, the molecular weight of PLGA gradually decreases while the overall weight remains stable, resulting in a decrease in mechanical strength in the first stage. In the second stage, the chain structure of the PLGA is destroyed, and the PLGA fragments are hydrolyzed into soluble molecules, manifesting a rapid decrease in the whole weight.^[^ ^53^, ^54^ ^]^ Besides, Figure 3d exhibits a thickness‐dependent degradation rate of ≈0.6 wt.%/day for PLGA and ≈0.11 wt.%/day for PLGA/PVA/PLGA in 7 days, which enables a predictable, controllable degradation process and could adapt to requirements of perioperative. The solubility of PEDOT: PSS is at a linear rate of 5.33 µg/(L⋅day), evaluated by results of inductively coupled plasma‐optical emission spectrometry (ICP‐OES, Figure 3e). The permeation of water into PEDOT: PSS weakens the ionic bonds between PEDOT oligomers and PSS chains Hydrophilic PSS chains accumulated on the surface and dissolved in water. Eventually, the PEDOT and PSS chains reorganized, and PEDOT: PSS no longer exhibit electrochemical regulation properties (Figure S10, Supporting Information).^[^ ^55^, ^56^, ^57^ ^]^ The solubility of Au is in an extremely low but stable range of 0.88–1.74 µg L⁻^1^ in continuous monitoring for seven days, which is consistent with the general knowledge of extremely low solubility and sluggish dissolution kinetics of metallic Au in solution. The insulation of interconnection suggests that Au was covered by PLGA insulating layer, so Au element in solution comes from the exposed Au connection pads. The possibility change in weight may cause by the formation and delamination of oxide or hydroxide species on the surface of Au, and it can be described by the function^[^ ^58^ ^]^:

(2) $$xAu+mH_{2}O⇌Au_{x}O_{m}H_{2m-n}^{xz-n}+nH^{+}+xze^{-}$$

Biodegradable metals, such as magnesium, molybdenum, and tungsten, can be introduced to replace Au in future research. However, challenges in biocompatibility and degradation speed need to be resolved.^[^ ^59^ ^]^ In addition, the electrical performance of electrochemical impedance (Figure 3f) increases, resulting from hydration of PEDOT: PSS and transformation from the quinoid structure to the benzoic structure with poor conductivity.

Furthermore, the effect of cross‐linked levels of PEDOT: PSS on performance decay was systemically investigated. The doping ratios of the thermal crosslinker of (3‐glycidyloxypropyl) trimethoxy silane (GOPS) were set as 0, 0.1, 0.5, 1.0, and 10.0 wt.%. Ethylene glycol (to improve the film conductivity) and 4‐dodecylbenzene sulfonic acid (DBSA, to enhance the film homogeneity) were kept consistent. A high temperature of 90 °C water bath (1X PBS solution, pH = 7.2) experiments were carried out to simulate the accelerated degradation process. The transfer curves of 30 samples were recorded at intervals of 5 min for each group. An interesting but unexpected result was obtained. All samples failed when they underwent a total of 45 min of immersion in 90 °C solution, showing degradation time characteristics independent of cross‐linker ratios. From this point of view, 45 min of accelerated degradation at 90 °C is ≈6–7 days at room temperature. However, the higher the degree of cross‐linking, the slower the device performance decays. At this time, the drain current and gate current were basically equivalent, both in microampere (µA) level, and the drain current was no longer electrochemically regulated by the gate voltage. All these results indicated that the microstructure of PEDOT: PSS is irreversibly damaged during the accelerated degradation. Water penetration into PEDOT: PSS bulk transformed the quinoid structure to the benzenoid structure with poor conductivity, resulting in a decrement in on‐state current and transconductance value.^[^ ^55^, ^56^, ^57^ ^]^ What can be predicted is that the degree of crystallinity is higher, and the device is more stable as the proportion of GOPS crosslinker increases. However, excessive GOPS limits the electrical properties of OECT due to its insulating properties. Taking the transconductance values as evaluation, the doping ratio of 1.0 wt.% achieves the best balance between stability and electrical properties (Figure 3g and Figure S11, Supporting Information). Similar stability correlations were observed for the on‐state currents (Figure 3h and Figure S12, Supporting Information). The degradation of the off‐state current of OECTs with various crosslinker ratios could be neglected, and no significant correlation was observed (Figure S13, Supporting Information).

### 2.3 In vivo characterization

**Figure 4:**
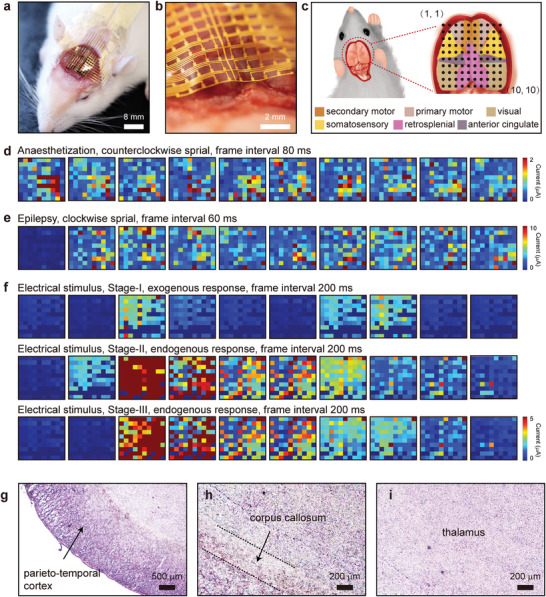
Characterization of OECT array degradation process Disintegration of layered microstructures in a transient electronic device during dissolution. a) Photograph of OECT array laminating onto the cerebral cortex of SD rat. b) Photograph of front view of the OECT platform seamlessly attached on the brain of a rat, illustrating the tissue‐compatible mechanical properties. c) Schematic diagram of the division of functional areas, and the OECT biosensors’ locations on the brain atlas. d) μ‐ECoG signals under anesthesia state, movie frame interval 80 ms; e) μ‐ECoG signals during seizures, movie frame interval 60 ms; f) μ‐ECoG signals with electric stimulus at 1 Hz and 3 V. Hematoxylin‐eosin staining results of the brain slice: Optical micrograph image of g) the edge of brain slice, marked as parieto‐temporal cortex; h) the middle of the brain slice, marked as corpus callosum; and i) the image of the core region of rat's brain, marked as thalamus.

In vivo μ‐ECoG signals recording experiments involved the biodegradable OECT array in 4–6 weeks‐old Sprague Dawley (SD) rats weighing 200–400 g, which was placed at the 8 × 8 mm^2^ region cortex (**Figure** **4**a). Given the high flexibility, the OECT array could softly and intimately laminate onto the brain without trauma (Figure 4b). The distribution of 100 units is displayed in Figure 4c. The positions are arranged along the coordinate set by a reference point to ensure all OECTs are in the same place in every experiment. When the brain is active, post‐synaptic potentials occurring simultaneously in a large number of neurons are summed to form brain waves, the essence of which is the movement of charged ions in and out of neuro‐synapses.^[^ ^60^ ^]^ The μ‐ECoG records the overall reflection of the EP activity of brain nerve cells in the cerebral cortex. In this case, the recording occurs by laminating the device's exposed PEDOT: PSS channels directly to the cerebral cortex. The cerebral cortex replaces the Ag/AgCl as gate input, and its potential changes with respect to a ground contact leads to drain currents’ transient fluctuation during the in vivo measurements. Similar methods and their feasibility have been validated.^[^ ^45^ ^]^ We recorded varied spatiotemporal μ‐ECoG signals patterned from 100 OECT units under three different situations of the SD rats, under anesthesia (Figure 4d), during the epileptic seizure (Figure 4e), and with the electrical stimulus to two hind legs (Figure 4f), and the corresponding sequences of movie frames are listed. It can find that Ketamine‐induced spikes rotate counterclockwise spiral with a speed of 90–120 ms per revolution and have signal amplitudes of ≈2 µA. Given the multi‐channels on the array and desirable time resolution, data from all channels is displayed for a typical sleep‐like spindle with a 10 ms frame interval (Figure S14, Supporting Information). Pentylenetetrazol was applied typically to induce epilepsy activity in which the drug was injected intravenously into the abdomen of rats, and the epilepsy spikes were recorded when the rats’ seizure behavior occur usually 30 mins later. Different from spikes in the anaesthetization state, pentobarbital‐induced epilepsy spikes show a spiral trend of clockwise spiral and greater peak amplitude with a speed of 130–140 ms per revolution (Figure 4e and Figure S15, Supporting Information).

For the third demonstration of this high‐throughput transient OECT array, we introduced alternating current (AC) electric stimuli on the hind leg of SD rats. Clear and exciting exogenous and endogenous responses evoked by electric stimuli were observed (Figure 4f‐I and Figure S16, Supporting Information). Movie frame‐I maps the exogenous response on the electric stimulation with 1 Hz during one second, and the evoked spikes show the same frequency of 1 Hz. A larger amplitude of the μ‐ECoG spike appears due to the stress reaction produced for self‐protection at the moment of stimulus. Then, it was manifested as a stable and uniform μ‐ECoG signal for the next 15 s.^[^ ^61^, ^62^ ^]^ That is the same story for electric stimuli with 3 Hz only on the left hind leg (Figure S17, Supporting Information). In addition, responses in the left and right brain's sensory and motor cortices responded were visible when two hind legs were electrically stimulated (Figure S16, Supporting Information). Only right brain respond when the left hind was electrically stimulated (Figure S17, Supporting Information). The successful of somatosensory evoked potential verified that the left brain controls the right limb, and vice versa. There were two endogenous responses when the electric stimuli terminated, which appeared in time windows of 1s (Figure 4f‐II and Figure S18a, Supporting Information) and 4s (Figure 4f‐III and Figure S18b, Supporting Information) that may result from the activation of the locus coeruleus and were followed by the activation of the sympathetic‐adrenomedullary system, hypothalamic‐pituitary‐adrenal axis. It is usually accompanied by significant changes in heart rate, blood pressure, muscle tension, and metabolism to deal with emergencies.^[^ ^63^ ^]^ Furthermore, understanding the immune response resulting from device rigidity and material toxicity is essential for clinical medicine applications. Studies of tissue immune reactions of transient array involved 30 days implants in experimental SD rats. Figure 4g–i summarizes the result of biocompatibility and immune response evaluated by in vivo the experiments of OECT array implanted subcutaneously into rats. In the hematoxylin and eosin (H&E) image, it can be observed parietal‐temporal cortex (Figure 4g), corpus callosum (Figure 4h), and thalamus (Figure 4i) in order from the outside in. No neutrophils, lymphocytes, and macrophage cell gathering was observed, indicating that acute inflammation did not occur or has been eliminated.^[^ ^64^ ^]^ Acute inflammation is associated with a response to an external stimulus, such as surgical incisions, which usually appear in the initial implantation stage.^[^ ^65^ ^]^ No granular tissue was observed in the H&E image, meaning that brain tissue did not wound at the time of surgery or during implantation. Therefore, this OECT platform can be used as a biocompatible implant without any side effects caused by the by‐products.^[^ ^66^, ^67^ ^]^

**Figure 5:**
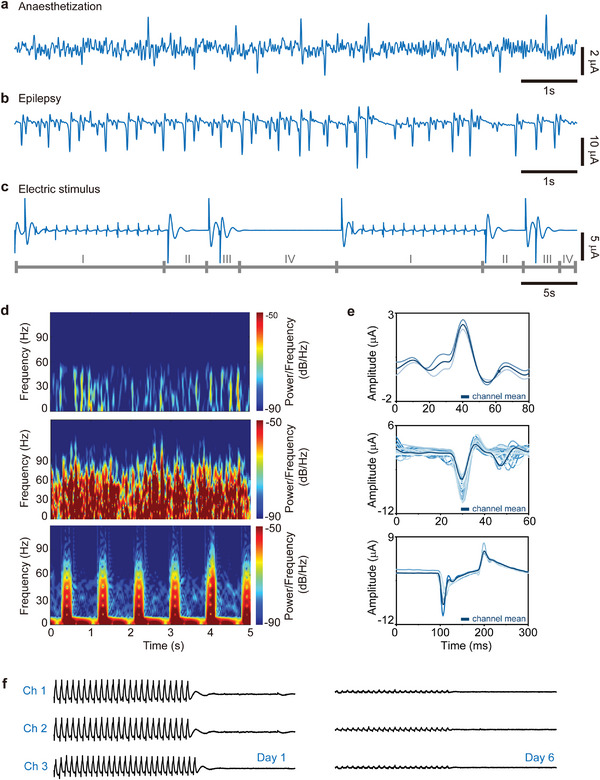
Characterization of μ‐ECoG signals for three representative stimuli. Time‐domain signals of rat a) in state of anaesthetization; b) during seizures; c) with electric stimulus at 1 Hz and 3 V. d) Time‐frequency‐power/frequency analysis during a short period, recorded in situations of anaesthetization (top), epileptic seizure (middle), and electric‐stimulus (bottom). e) Associated characteristic evoked waveform anaesthetization (top), epileptic seizure (middle), and electric stimulus (bottom), where dark blue lines are channel mean. f) Representative μ‐ECoG signals recorded by the transient OECT array on day 1 and day 6, and recordings from three channels in array exhibit large‐scale spikes induced by electrical stimuli at 3 V and 3 Hz that consistent with the related local and temporal variations.

**Figure** **5**a–c shows representative activities captured by one of the channels in the transient biodegradable OECT array. The origin and interpretation of the cortical waves under different brain states are different, which can be distinguished by signal amplitude, waveform, and frequency. The μ‐ECoG signal during anesthesia displays both K‐complexes and sleep spindle waveforms with low amplitude and frequency characteristics. The μ‐ECoG signals during the epilepsy activity reveal a sizeable negative peak amplitude of 9.8 µA, low noise of 0.63 nA, and SNR of 23.8 dB, which has a similar brain waveform with previous reports at the ictal onset.^[^ ^7^ ^]^

The high‐fidelity recordings of μ‐ECoG and evoked potentials in the demonstration of electric stimuli are consistent with the movie frames in Figure 4f. It has been divided into four stages: stable exogenous response (stage‐I), the first endogenous response (stage‐II), the second endogenous response (stage‐III), and quiet period (stage‐IV), revealing the propagation of neural spikes and associated spatiotemporally resolved patterns. The neurons then stopped firing and returned to calm until the next stimulus came. The exogenous μ‐ECoG signals in this situation exhibit a large peak amplitude of ∼ ± 12 µA, low noise of 16.4 nA, and high SNR of 37.0 dB. Figure S19a–c (Supporting Information) compare the waveforms of exogenous and endogenous responses, and Figure S19d,e (Supporting Information) compare the waveforms of exogenous response under electric stimuli at 1 Hz and 3 Hz. We also performed light stimulation experiments in which the electrical activity in the visual and secondary motor areas of the rat cerebral cortex was enhanced with increased amplitude when UV light was directed to the right eye of the rats. In contrast, the rest of the cerebral cortex barely responded (Figure S20, Supporting Information). Frequency‐domain characterization analysis of various stimuli revealed the presence of typical oscillations, as is shown in Figure 5d. Remarkable differences during recordings were observed in anaesthetization stimuli (top) with low‐amplitude oscillations below 10 Hz, epilepsy stimuli (middle) with high‐amplitude oscillations up to 60–70 Hz, and in electric stimuli (bottom) with high‐amplitude oscillations at 1 Hz. In addition, a set of characteristic evoked spikes is shown in Figure 5e as the representative recordings under the three stimuli, including onset time window, signal amplitude, and waveform, which have been summarized in Table S4 (Supporting Information). The abovementioned results demonstrate that this high‐throughput transient neural interface can capture reliable physiological or pathological activity from the cerebral cortex in animal models. Figure 5f and Figure S21 (Supporting Information) summarize representative cerebral cortex potentials recorded by three channels in the transient neural interface. The electric stimulus of 3 V (3 Hz and 2 Hz) were applied within six days after the surgical implantation. Results reveal that the μ‐ECoG signals faded in spacetime throughout one week of the study, due to the degradation of PEDOT: PSS, which is consistent with the findings in vitro. Currently, the PLGA substrate and insulating layer are intact without any cracks, as well as the source‐drain electrodes. For patients with cardiovascular surgery, a perioperative period of 48 h is most critical, while it may take longer time for epilepsy patients, which requires to encapsulate of this OEDOT: PSS‐based neural interface or replacement with more electrochemical stable semiconductors.

## 3 Conclusion

The multichannel, transient OECT platforms introduced here exploit ultrathin, soft architectures that are capable of laminating onto the cerebral cortex seamlessly as the monitoring systems for high‐fidelity recording of various μ‐ECoG signals, given their tissue‐compatible mechanical properties and desirable electrical performance. Such a high‐resolution transient platform, facilitating mapping and elucidating the mechanisms of captured μ‐ECoG signals, is significantly distinguished in form and function over previous efforts on biodegradable neural interfaces. Demonstrations in situations of anaesthetization, epileptic seizure, and electric stimulus in rat models are representative of a broad spectrum of potential short‐term clinical applications, such as monitoring for early signs of failure during critical postoperative periods and recording for the study of cognitive function and artificial intelligence. Systematic studies on materials science, electrics, and neuroscience in this OECT platform provide a basic understanding and guidelines for device design and aim to form a robust foundation of the capabilities in implantable transient electronics technology for various biomedical applications.

## 4 Experimental Section

### OECT Fabrication

Fabrication began with growth of 12 µm of PLGA (65:35, 20 wt.% dissolved in chloroform) substrate onto clean glass slides. The films were baked at 160 °C for 20 min and then 100‐nm‐thick Au films were thermally evaporated. Au electrodes were patterned with the aid of AZ4620 photoresist and etching by a mixed solution of iodine (I_2_) and potassium iodide (KI). Polyvinyl alcohol (PVA, 4 wt.% dissolved in DI water) was spin‐coated onto Au to separate substrate and insulating layer. Another 100 nm of PLGA layer was spin‐coated with a concentration of 5 wt.% reserving as insulating layer. Then these films were patterned with successive photoresist and reactive ion etching by an O_2_ plasma (120 W, 20 sccm, 12 min) using a Plasmatherm 790 Reactive Ion Etch Machine. For the preparation of PEDOT: PSS films, 10 mL of aqueous dispersion (Clevios PH1000) was mixed with 5 wt.% ethylene glycol, 0.25 wt.% DBSA, 1.0 wt.% (3‐glycidyloxypropyl) trimethoxysilane (GOPS, acting as the crosslinker), and the resulting dispersion was spin‐coated at 1500 rpm and baked at 120 °C for one hour. In the study of the relationship between crosslinking level and device degradation, the GOPS were 0, 0.1, 0.5, 1.0 and 10 wt.%, respectively, while PEDOT: PSS aqueous dispersion, ethylene glycol, DBSA were kept constant mixture ratios. Followed by a similar etching process to pattern the PEDOT: PSS to a size of 200 × 20 µm^2^. More detailed device fabrication can be found in Figure S3–S6 (Supporting Information).

### Device Characteristics

The transistors were characterized in vitro using 1X PBS solution as the electrolyte. And the Ag/AgCl were applied to as the reference electrode. To characterize the OECTs, the source, drain and gate electrodes were connected to a semiconductor analyzer (Keysight B1500A), where the source electrode was grounded. Both output and transfer characteristics of OECT were obtained via the IV measurement software available from Keysight B1500A. To measure output characteristics, a gate‐source potential difference was applied from 0 to 0.6 V in 0.1 V steps and the drain voltage was swept from 0 to ‐0.6 V for each step during which drain current was measured. To measure transfer characteristics, the drain potential was kept constant at ‐0.5 V and drain current was measured. The gate voltage was swept from 0 to 0.5 V and gate current was also measured. To measure response time, the drain voltage was kept constant at ‐0.5 V and a voltage step was applied to the gate from ‐0.2 to 0.6 V in the form of a square wave. Then the drain currents were sampled. To characterize transconductance‐frequency, the gate was driven by various sin waves accordingly provided by a Signal Generator/Signal Source (Keysight). The impedance spectrum of PEDOT: PSS was conducted with electrochemical workstation (CHI 660E) from 100K Hz to 0.1 Hz. Substrate thickness was measured by the Bruker Dektak XT Profilometer. Sheet resistance was measured by Four Point Probe (DMR‐10). The weight of films and OECT devices were evaluated by high‐precision analytical electronic balance with an accuracy of 0.1 mg (MeilenMCS220), and the ICP‐OES resulted were obtained from the professional testing organizations (Easy Analysis Technology (Guangzhou) Co.).

### In Vivo Evaluation

All procedures were conducted in accordance with the ethical guidelines of the Department of Health of Kong Hong, animal studies and the experimental protocol was reviewed and approved by the Institutional Animal Care and Use Committee at the City University of Hong Kong (approval number A‐0664), involved anaesthetized 4–6 weeks old male SD rats with their head fixed in a stereotaxic apparatus. First, the rats were weighted and anesthetized with ketamine by intraperitoneal injection (0.35 mg ketamine for 1 g weight of rats). Then, the hair on rat's head was removed with depilatory cream. After 10–15 min of anesthesia injection, the rats were in a state of deep anesthesia. At this time, the scalp of the rat was subtracted with sterilized surgical scissors to expose the skull. And then the mixed gas of isoflurane and oxygen (1%‐3%, RWD Life Science Co., Ltd, R640 Light and Simple Animal Anesthesia Machine) was rapidly induced and recovered by a trachea inserted into the rat's mouth. Heat rate and blood pressure were monitored throughout the experiments to ensure depth and stability of anesthesia. Body temperature was maintained with a heating blanket at 37 °C during the whole procedure process, and the anal temperatures of the rats were in the range of 35–37 °C. The craniotomy procedure was performed with a hand‐held electric drill (RWD Life Science Co., Ltd, 78 001), and the diameter of the cranial drill bit was ≈0.5 mm (RWD Life Science Co., Ltd, HM1005). After removing the dura, The OECT array was placed onto the exposed cerebral cortex in the region of 8 mm × 8 mm, and a slurry of gel‐foam and saline was layered on top of the OECT platform. A screw electrode was placed posterior to lambda as a ground electrode, and additional screws were secured in the skull for anchoring. The skull and OECT platform were then covered with dental cement and the connecting plug was secured on top. For relieving postoperative pain, the rats were given meloxicam daily. After three days, the rats were used for neural signals recording.

### Chronic Evaluation of Immunohistochemistry

Rats were anaesthetized and exposed in CO with a concentration of 0.2%‐0.5%. After the rats died, the brains were then removed and post‐fixed overnight in 4% paraformaldehyde solution at 4 °C, followed by cryoprotected in 30% sucrose for three days. Coronal sections were cut at 20 µm using a cryostat (Cryostar NX70 motorized Cryostat with vacutome). Serial sections for the entire craniotomy site were mounted onto the changed slides and stored at – 20 °C before use. For immunostaining, the slides were first rinsed in distilled water, incubated in hematoxylin stain for 5 min at room temperature, and then washed with distilled water for 5 min. Then slides were differentiated with the 0.1% mixed solvent of hydrochloric acid and ethanol for 1–3 s, followed by washing with distilled water for 1 min. Next, the slides were detained in 1X PBS solution for 30 s, followed by washing with distilled water for 3 min. Then, the slides were rinsed in 95% ethanol for 5–10s and counterstained with eosin staining for 30 s. Finally, the slides were dehydrated twice with ethanol for 3 min, and cleared in dimethylbenzene twice for 3 min. These slides were examined on an optical microscope and images were acquired with a 20× objective.

### Data Acquisition

The prepared OECT was connected to a self‐developed printed circuit board (PCB) board via a commercial ACF cable. This PCB provided driving signals with the multiplexer of ADG725BCPZ at sampling rate at 1000 Hz every line. And code composer studio was used for circuit board programming. OECT output a current, while multiplexer only could collect the voltage data. Therefore, a 10‐ohm resistor was connected in series at the output end of each channel, and the current of the corresponding channel could be obtained by recording the voltage value across the resistor. Detailed circuit diagram design and PCB design can be found in Figures S22–S24 (Supporting Information). Data were acquired by the PowerLab data acquisition (PL3516/P PowerLab 16/35, AD Instruments) with sampling frequency of 20000 Hz. To ensure the reliability of the connection of the OECT array to PowerLab, the transfer characteristics of the OECT array in 1X PBS solution with Ag/AgCl gate were measured. And animal experiments were performed when all OECT units worked. The μ‐ECoG data for all experiments were filtered using a fourth Butterworth bandpass filter in the forward direction to obtain zero‐phase distortion digital filtering, and then processed by customized programs in MATLAB R2022a.

## Conflict of Interest

The authors declare no conflict of interest.

## Supporting information

Supporting Information

## Data Availability

Research data are not shared.
