# Supplementary material for: Ultrathin, Soft, Bioresorbable Organic Electrochemical Transistors for Transient Spatiotemporal Mapping of Brain Activity
Source: Adv Sci (Weinh). 2023 Feb 24;10(14):2300504. doi: 10.1002/advs.202300504 (PMC10190644; doi:10.1002/advs.202300504)
Supplement: Supplementary file 1 — Supporting Information [file ADVS-10-2300504-s001.pdf]

Supplementary Materials

Ultrathin, soft, bioresorbable organic electrochemical transistors for transient spatiotemporal mapping of brain activity

Mengge Wu [a,b,c], Kuanming Yao [b], Ningge Huang [c], Hu Li [b], Jingkun Zhou [b,d], Rui Shi [b], Jiyu Li [b,d], Xingcan Huang [b], Jian Li [b,d], Huiling Jia [b,d], Zhan Gao [b], Tsz Hung Wong [b], Dengfeng Li [b,d], Sihui Hou [a,b], Yiming Liu [b], Shiming Zhang [e], Enming Song [c *], Junsheng Yu [a *], Xinge Yu [b,d *]

Affiliations:

[a] State Key Laboratory of Electronic Thin Films and Integrated Devices, School of Optoelectronic Science and Engineering, University of Electronic Science and Technology of China (UESTC), Chengdu 610054, China. Email: jsyu@uestc.edu.cn

[b] Department of Biomedical Engineering, City University of Hong Kong, Hong Kong, China. Email: xingeyu@cityu.edu.hk

[c] Shanghai Frontiers Science Research Base of Intelligent Optoelectronics and Perception, Institute of Optoelectronics, Fudan University, Shanghai 200433, People's Republic of China. Email: sem@fudan.edu.cn

[d] Hong Kong Center for Cerebra-Cardiovascular Health Engineering, Hong Kong Science Park, New Territories, Hong Kong, China

[e] Department of Electrical and Electronic Engineering, The University of Hong Kong, Hong Kong SAR, China

a

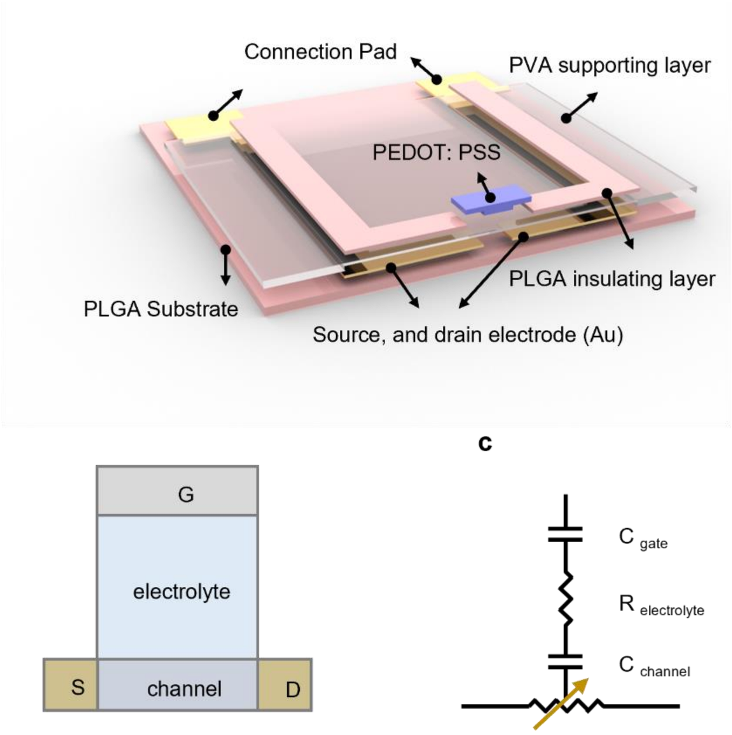

Supplementary Fig. S1. **a**, Explored view of single OECT device. **b**, Ionic circuit of OECT. **c**, Electronic circuit of OECT.

Note:

The structure of OECT is displayed in Fig. S1a. OECT possesses ultrahigh transconductance values, on the order of milliSiemens for micrometer-scale devices, at least two orders of magnitude higher than that of FET, suggesting higher amplification ability and lower detection of limit. This can be attributed to that the ions from electrolyte can penetrate into the whole body of the channel and induce a volumetric nature of their response, while response process only happens on dielectric/channel interface in field-effect transistors (Figs. S1b and 1c). Furthermore, OECT can conduct both ionic and electronic current, enhancing the efficiency and decreasing the electrochemical impedance mismatch of the electrode with brain tissue.

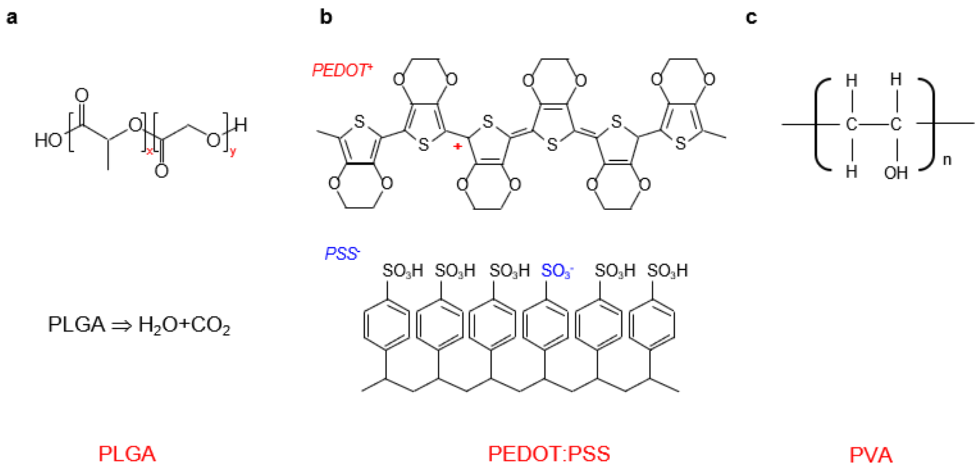

Supplementary Fig. S2. Chemical structures used in the flexible, ultrathin, high-throughput, transient OECT array. **a**, PLGA, where x refers to the number of units of lactic acid and y refers to the units of glycolic acid. And PLGA can decompose into biosafety water and carbon dioxide. **b**, PEDOT: PSS, a polymer mixture of two ionomers of $PEDOT^+$ and PSS^-. **c**, PVA, a water-soluble synthetic polymer and has the idealized formula $[CH_2CH(OH)]_n$.

Note:

PEDOT: PSS is important to be the channel material for OECT can be attributed to three reasons: (i) Excellent biocompatibility and bio-absorbability. [1] This can avoid biological reactions, such as blood, immune, and tissue reactions, caused during in vivo applications. Meanwhile, it also ensures that the device is not affected by the internal environment and can still work properly. (ii) The outstanding electrochemical performance of PEDOT: PSS allows the ability to record and amplify the low amplitude neural signals in OECT. [2, 3] (iii) Scalability and commercialization.[4] The materials involved in PEDOT: PSS are commercially available, including PEDOT dispersions, crosslinkers, and additives, which ensure high reproducibility of PEDOT: PSS-based OECT. Meanwhile, spin-coating, spraying, and ink-jet printing methods for large-area PEDOT preparation are well established, paving the way for its commercial applications.

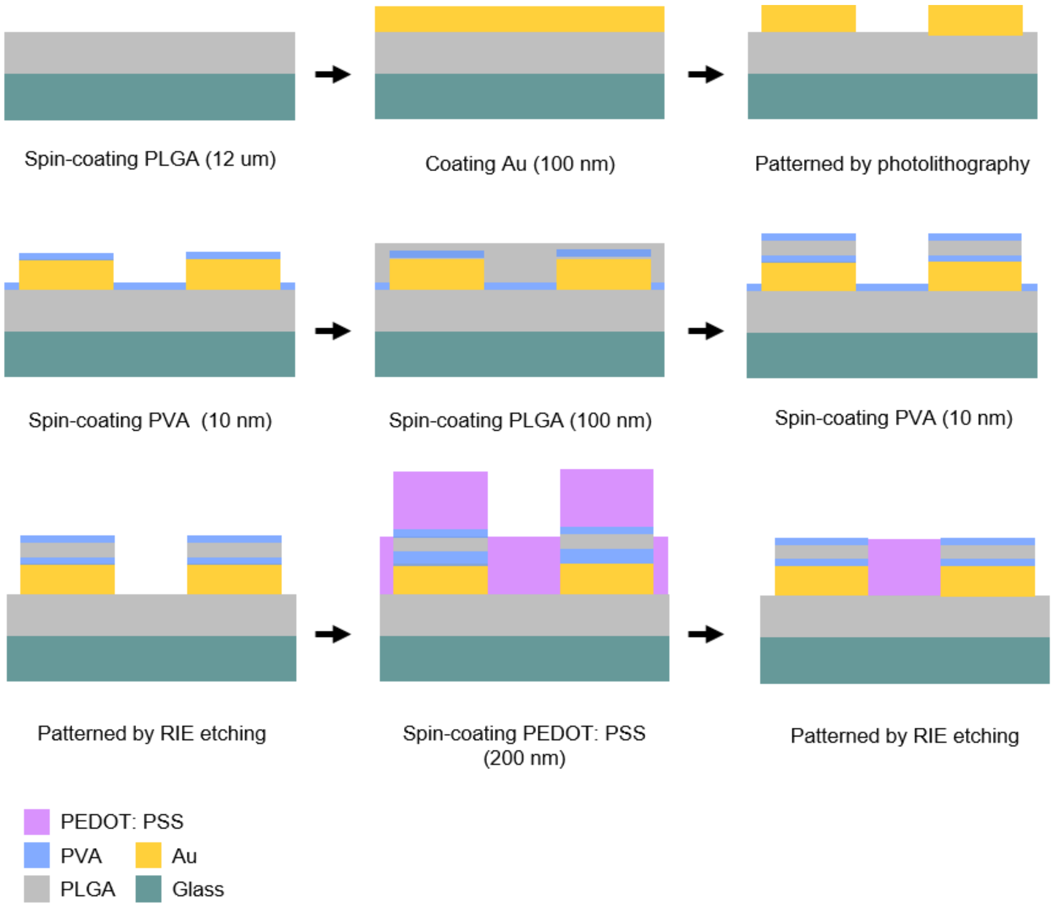

Supplementary Fig. S3. **Schematic illustration of the flexible, ultrathin, high-throughput, transient OECT array fabrication process.**

Before the experiment, glass substrates (75 mm × 75 mm × 1mm) were ultrasonically cleaned with detergent, acetone, and isopropanol in sequence. After drying the surface water with nitrogen, the glass substrates were treated with UVO for 15 min for use.

Step-1: Preparation of PLGA substrate. Spin-coating PLGA solution on the glass as the substrate of flexible OECT platform, at a speed of 1500 r.p.m. for 60 s, and then annealed on 160 ^{o}C hot plat for 20 min to remove the solvent. PLGA solution was prepared by dissolving the PLGA pellets (Sigma-Aldrich, lactide: glycolide =65:35, average Mn=6500) in chloroform, with a mass ratio of 20 wt.%.

Step-2: Preparation of Source-drain Electrode Array. About 100 nm-thickness Au layers were thermally deposited on PLGA/glass substrates. Spin-coating AZ4620 photoresist at the speed of 3000 r.p.m. for 30 s, and then these samples were baked at 115 ^{o}C for 5 min. Next, the above samples were exposed to 280 UV light (Mask Aligner model URE 2000) for 45 s, where a self-designed film lithography board (Mask-1, as shown in Figure S3) was covered on photoresist layers to block the passage of the UV light in the pattern region. After that, these samples were developed in AZ400 developer for 90 s to remove the exposed photoresist and remain the unexposed region, and then they were rinsed in DI water for 10 s to remove residual developer. Next, these samples were etched in Au-etching-solution (the mixed solution of potassium iodide and iodine)

for 60 s to etch away the Au that was not covered by the photoresist, and another washing process was performed to remove residual Au-etching-solution and clean the samples' surface. Finally, these samples were re-exposed using Mask Aligner where a blank lithography board was used to expose the whole surface of the samples, followed by a re-developing process for 3 min to remove all the photoresist on the patterned Au layer. Typically, the last step to remove the photoresist was conducted by acetone, however, the method of re-exposure and development was taken here. This is because that acetone could corrode the underlying PLGA substrate, while the photoresist and developer would not.

Step-3: Preparation of electrode encapsulation layer. To ensure the stable operation of the OECT and reduce the gate voltage drop at source-drain electrodes, encapsulation was necessary. At first, an ultrathin polyvinyl alcohol (PVA, Sigma-Aldrich, Mw approx. 30000) layer was spin-coated on the Au/PLGA/glass samples at the speed of 1500 r.p.m. for 20 s and baked at 100 ^{o}C for 10 min. PVA layer was introduced here as a protection between the encapsulation layer and the PLGA substrate, otherwise, the PLGA encapsulation layer to be spin-coated would dissolve the substrate. Then, the PLGA solution was spin-coated onto the PVA layer at the speed of 3000 r.p.m. for 15 s and baked at 160 ^{o}C for 20 min. PLGA solution was prepared via dissolving the PLGA pellets (Sigma-Aldrich, lactide: glycolide =65:35, average Mn=6500) in chloroform with a mass ratio of 5 wt.%. To improve the hydrophilicity of the surface of the PLGA encapsulation layer and enhance the uniformity of the PEDOT: PSS film, another identical PVA layer was spin-coated onto the PLGA encapsulation layer.

Next, patterning the PVA layer/PLGA encapsulation layer/PVA layer via the method of photolithography and reactive-ion etching (RIE). At first, spin-coating AZ4620 photoresist at the speed of 3000 r.p.m. for 30 s, and then these samples were baked at 115 ^{o}C for 5 min. Next, the above samples were exposed to 280 UV light (Mask Aligner model URE 2000) for 45 s, where a self-designed film lithography board (Mask-2, as shown in Figure S4) was covered on photoresist layers to block the passage of the UV light in the pattern region. After that, these samples were developed in AZ400 developer for 90 s to remove the exposed photoresist, and then they were rinsed in DI water for 10 s to remove residual developer. After drying with nitrogen gas, these samples were transferred into the RIE machine (Plasma-Therm 790 RIE). Oxygen was used to etch the PVA/PLGA/PVA layers for 5 min with a power of 120 W and case pressure of 120 mTorr. After the RIE process, patterned samples were obtained and the conductive channels region were exposed. Finally, these samples were re-exposed using Mask Aligner and developed with AZ400 to remove the top layer of photoresist.

Step-4: Preparation of conductive channels. In this work, thermal crosslinking PEDOT: PSS was utilized as active channel materials. PEDOT: PSS was prepared by mixing 10 mL of the aqueous dispersion (Clevios PH1000), 5 wt.% ethylene glycol (Sigma-Aldrich), 0.25 wt.% 4-dodecylbenzenesulfonic acid (Sigma-Aldrich) and 1 wt.% (3-glycidyloxypropyl) trimethoxysilane (Sigma-Aldrich). Before use, the mixed PEDOT: PSS solution was magnetically stirred for 48 hours. The PEDOT: PSS was spin-coated on the patterned samples with the structure of PVA/PLGA/PVA/Au/PLGA/glass at the speed of 1500 r.p.m. for 60 s and baked at 140

^{o}C for 1 hour. Next, PEDOT: PSS was patterned using the same set of photolithography procedures, including spin-coating AZ4620 photoresist, exposing to UV light (Mask-3, as shown in Figure S5), developing with AZ400 developer, RIE treatment with oxygen plasma, and finally removing the residual photoresist with the aid of re-exposure (blank lithography board) and re-development.

Step-5: Obtaining the flexible OECT array. Soaking the device fabricating by the above procedures in DI water for 1 hour to reduce the covalent bonding between glass and PLGA substrate. Then, use a tweezer to peel the device along the edge of the glass substrate. At this time, fully flexible, biodegradable OECT arrays were obtained.

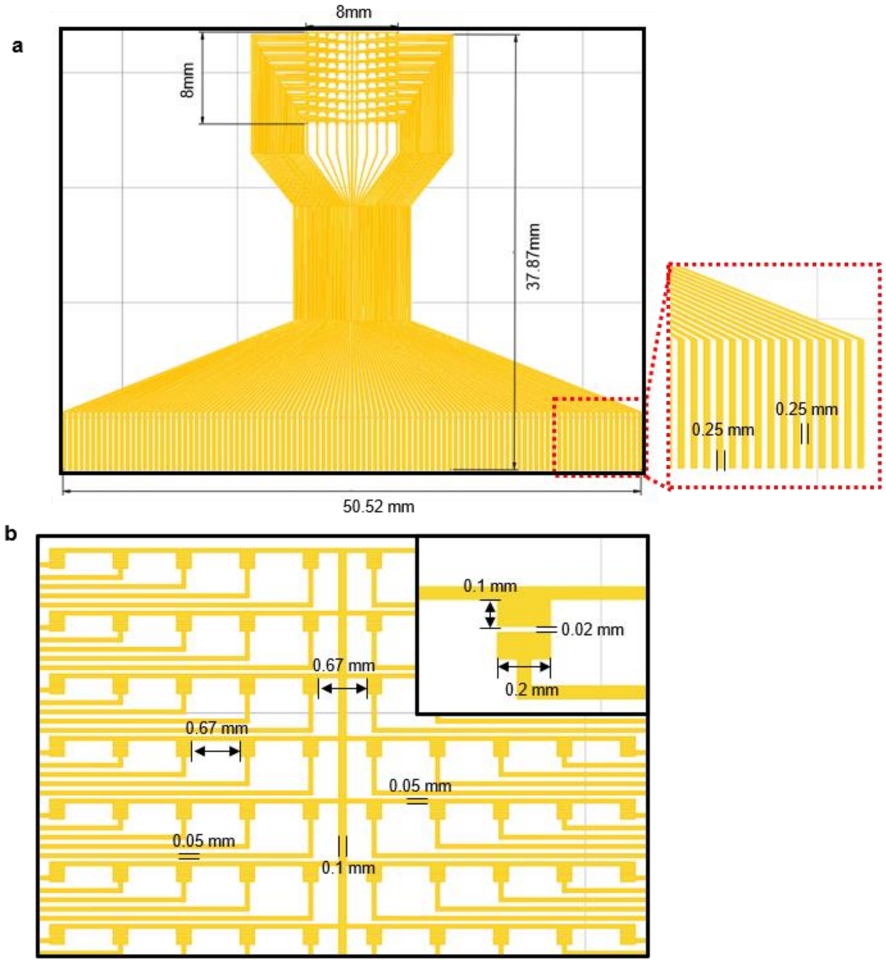

Supplementary Fig. S4. Mask-1 for patterning source-drain electrodes in OECT array. **a**, The overall writing design of source and drain electrodes in transient OECT array, where gold region blocks the passage of UV light and white region allows the passage of UV light. **b**, Amplified schematic diagram of the active sensing area.

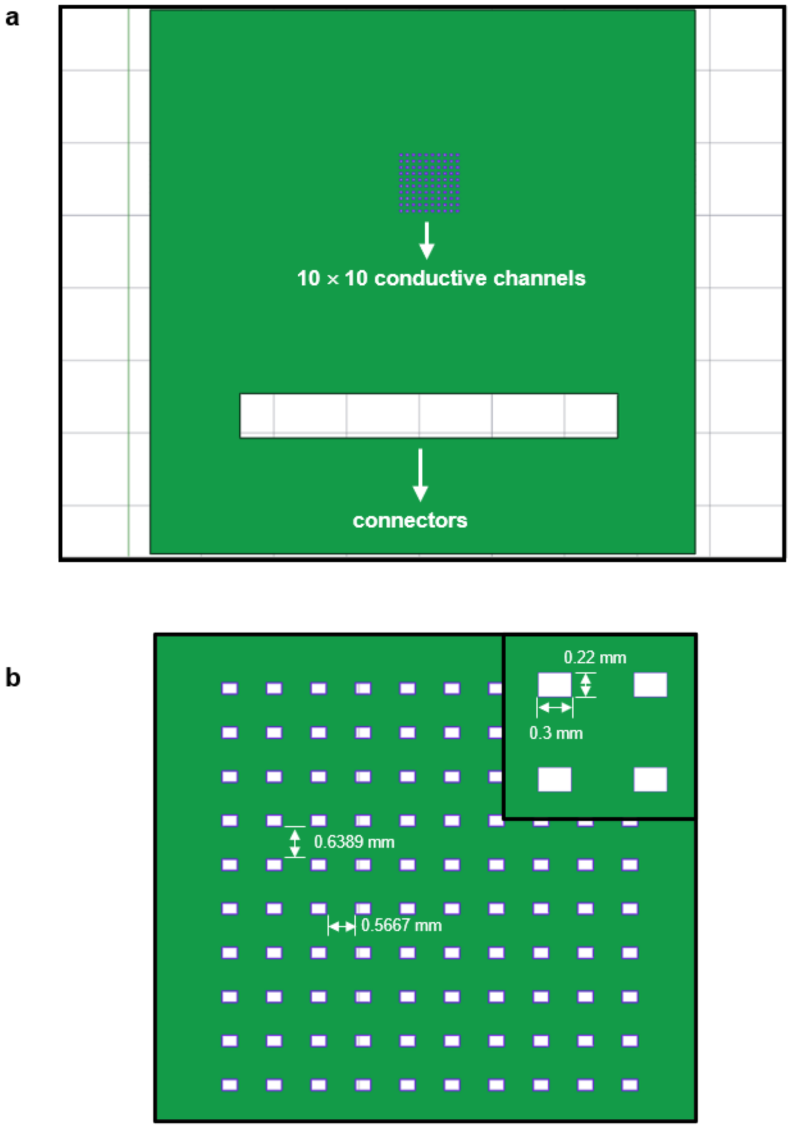

Supplementary Fig. S5. Mask-2 for patterning PVA/PLGA/PVA encapsulation in OECT array. a, The overall schematic of Mask-2, where green-color region blocks the passage of UV light and white-color region allows the passage of UV light. **b**, Amplified schematic diagram of the active sensing area.

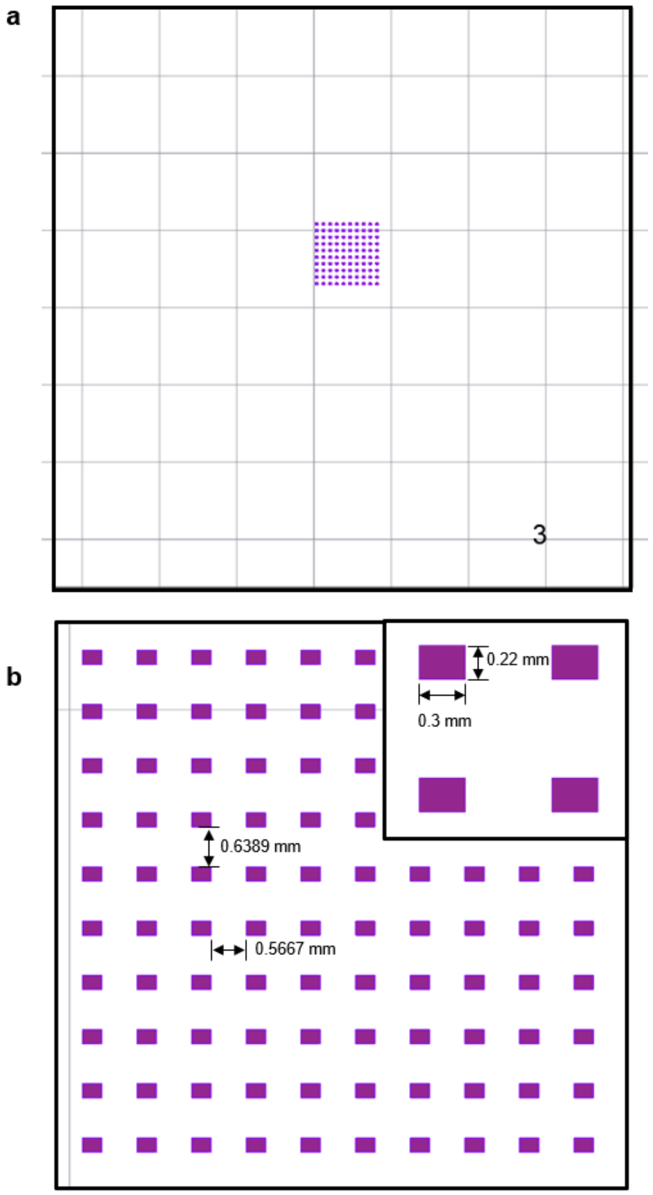

Supplementary Fig. S6. Mask-3 for patterning PEDOT: PSS in OECT array. **a**, The overall schematic of Mask-3, where purple-color region blocks the passage of UV light and white-color region allows the passage of UV light. **b**, Amplified schematic diagram of the active sensing area.

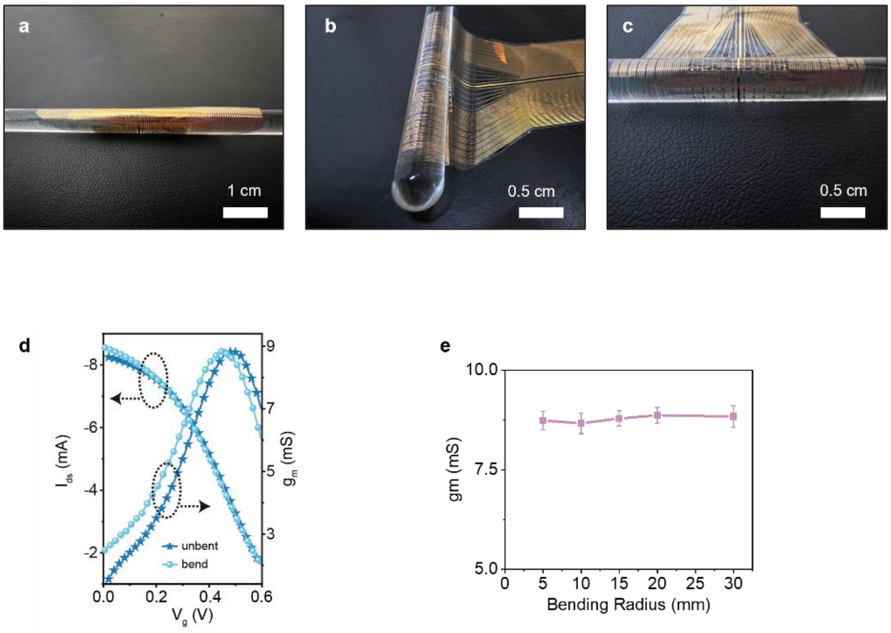

Supplementary Fig. S7 OECT array bends along a glass rod with a diameter of 5 mm. a, Front view of the device completely bends along the glass rod. **b**, Side view of the OECT array bend along the glass rod in one cycle. **c**, Front view of the OECT array bends along the glass rod in one cycle. **d**, Transfer and transconductance curves of the OECT under bend (shallow blue) and unbent states (dark blue).

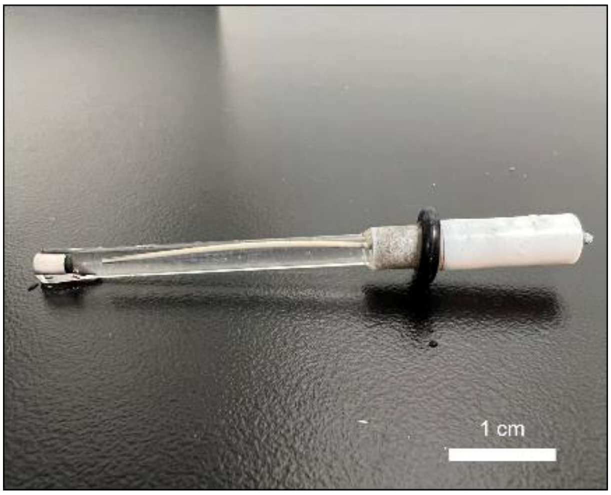

Supplementary Fig. S8 The optical image of Ag/AgCl reference electrode.

Note:

Ag/AgCl is a type of reference electrode, commonly used in electrochemical measurements. As shown in Figure S8, Ag/AgCl was brought from Shanghai Magyue Electronic Technology Co., and the product number is CH111. This Ag/AgCl electrode glass tube is 5 mm in diameter and 8 cm in length, with a sintered ceramic sand core at the bottom of the glass and filled with a saturated potassium chloride solution.

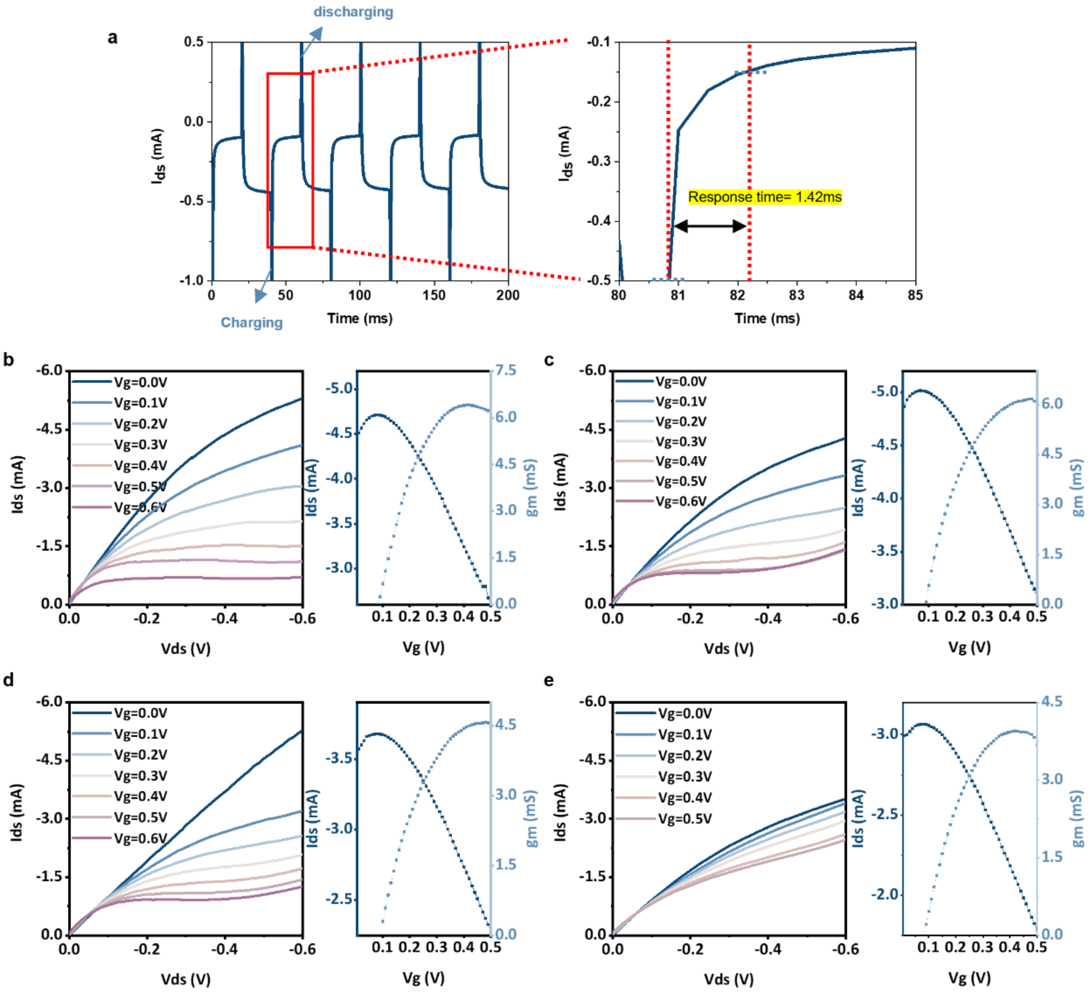

Supplementary Fig. S9. **a**, Study on response time of OECT unit. During the cyclic sweeping process, it can be found that the response speed of OECT is very fast with a response time of 1.42 ms. Even the charging and discharging behaviors have been recorded. Study on the degradation process of OECT: Output characteristic and transfer characteristic of **b**, Second day. **c**, Third Day. **d**, Fourth day and **e**, Fifth day.

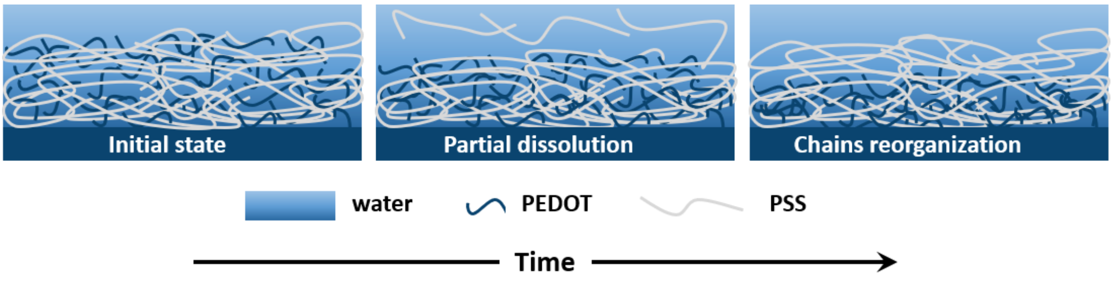

Supplementary Fig. S10. Schematic diagram of morphological changes of PEDOT: PSS film immersed in water with time.

Note:

PEDOT: PSS is not stable in PBS solution due to the phenomenon of hydration [5]. The permeation of water into PEDOT: PSS leads to the microstructure transformation and the ionic bonds breakage between the PEDOT oligomers and the PSS chains. The weight decreased as we observed with the rate of 5.33 μg/(L×day) since hydrophilic PSS chains accumulated on the surface of film and dissolved in water after the ionic bonds broke, which has been verified by the change in contact angle.

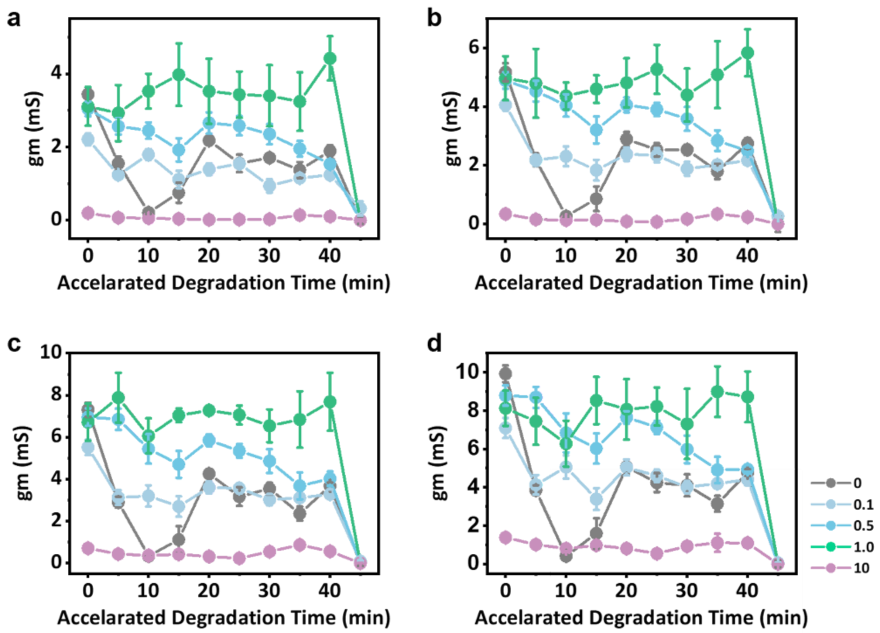

Supplementary Fig. S11. The relationship between the cross-linked level of PEDOT: PSS and degradation. a, gate voltage is 0.1 V. **b**, gate voltage is 0.2 V. **c**, gate voltage is 0.3 V. **d,** gate voltage is 0.5 V.

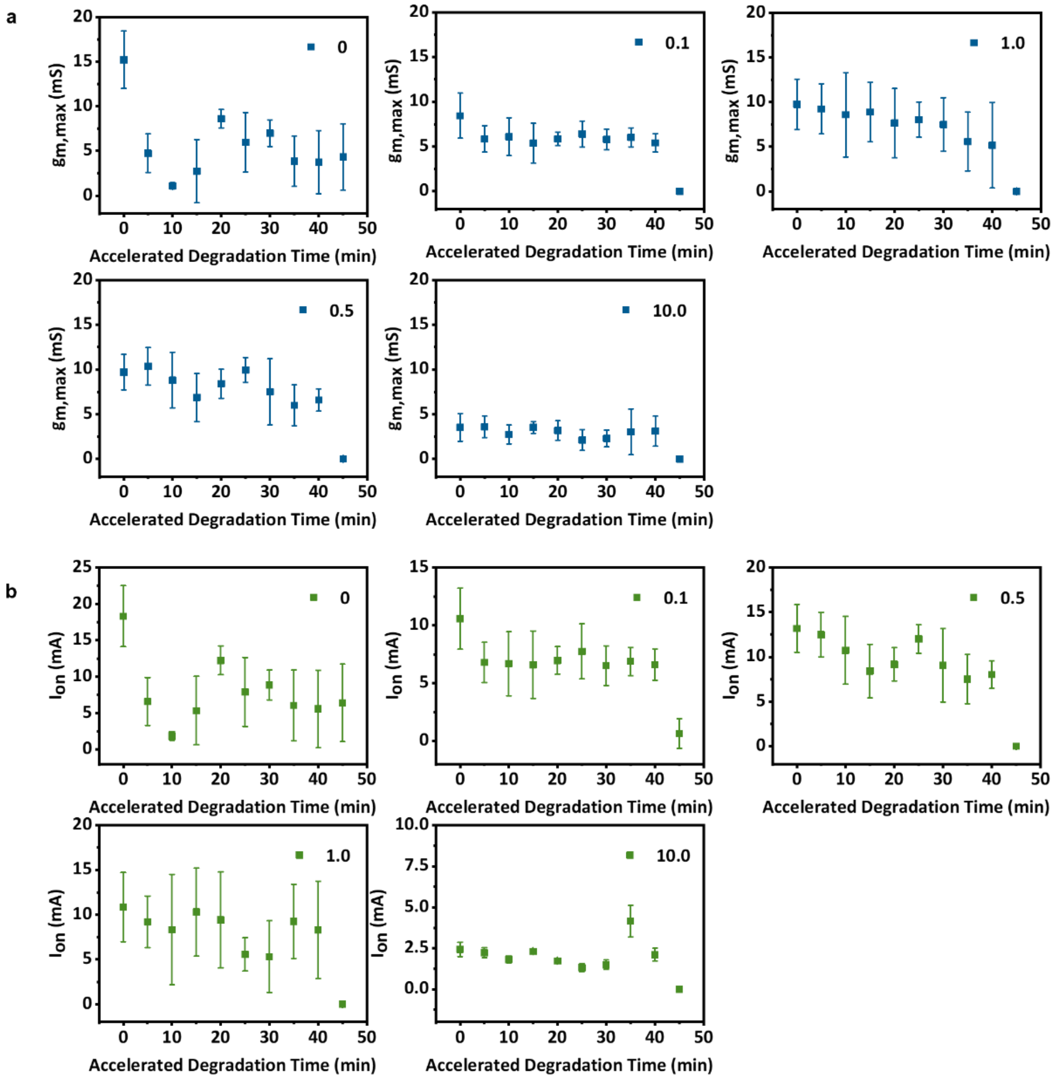

Supplementary Fig. S12. The relationship between the cross-linked level of PEDOT: PSS and degradation. **a**, change in $g_{m,\ max}$. **b**, change in on-state current.

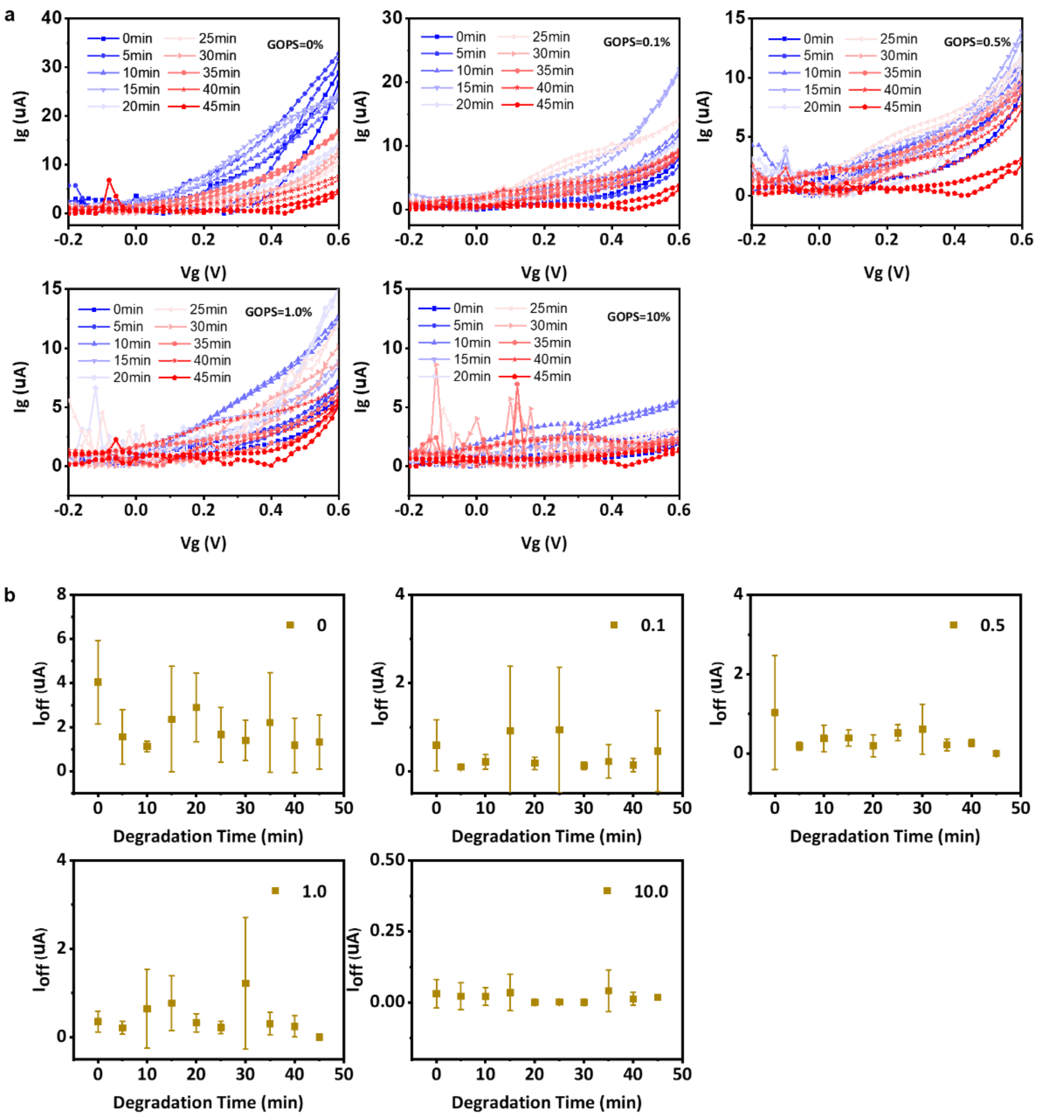

Supplementary Fig. S13. Study on the change in current during OECTs degradation process. a, gate current. **b,** off-state current.

Note:

To explore the relationship between gate current (i.e., leakage current) and device degradation, transfer characteristic curves during continuous high temperature accelerated degradation were conducted. At First, according to the equivalent electronic circuit and ionic circuit of OECT, there are two pathways for current flow, one is drain current that is electrochemically regulated by the change in gate voltage, and another is gate current that is generated by the potential drop between gate electrode and source electrode (ground electrode). Ideally, the effective gate voltage falls on the interface with channel so that the OECT can effectively regulate the drain current. However, the channel swelling even broken when the device underwent long-term immersion, which usually leads to two negative effects as follows. On the one hand, leading to channel impedance increases and on-state current decreases; on the other hand, leading to the fluctuation of gate current. It can be found that the gate current tends to increase first and then decrease. Gate increases probably because the source electrode is

exposed to the electrolyte and the voltage drop between gate and source increases during the device aging process. However, a reasonable explanation for the gate current reduction has still not been found. Overall, the change in gate current is in a small range of magnitude and can be ignored. Therefore, it can be concluded that there is no strong correlation between gate current and device degradation

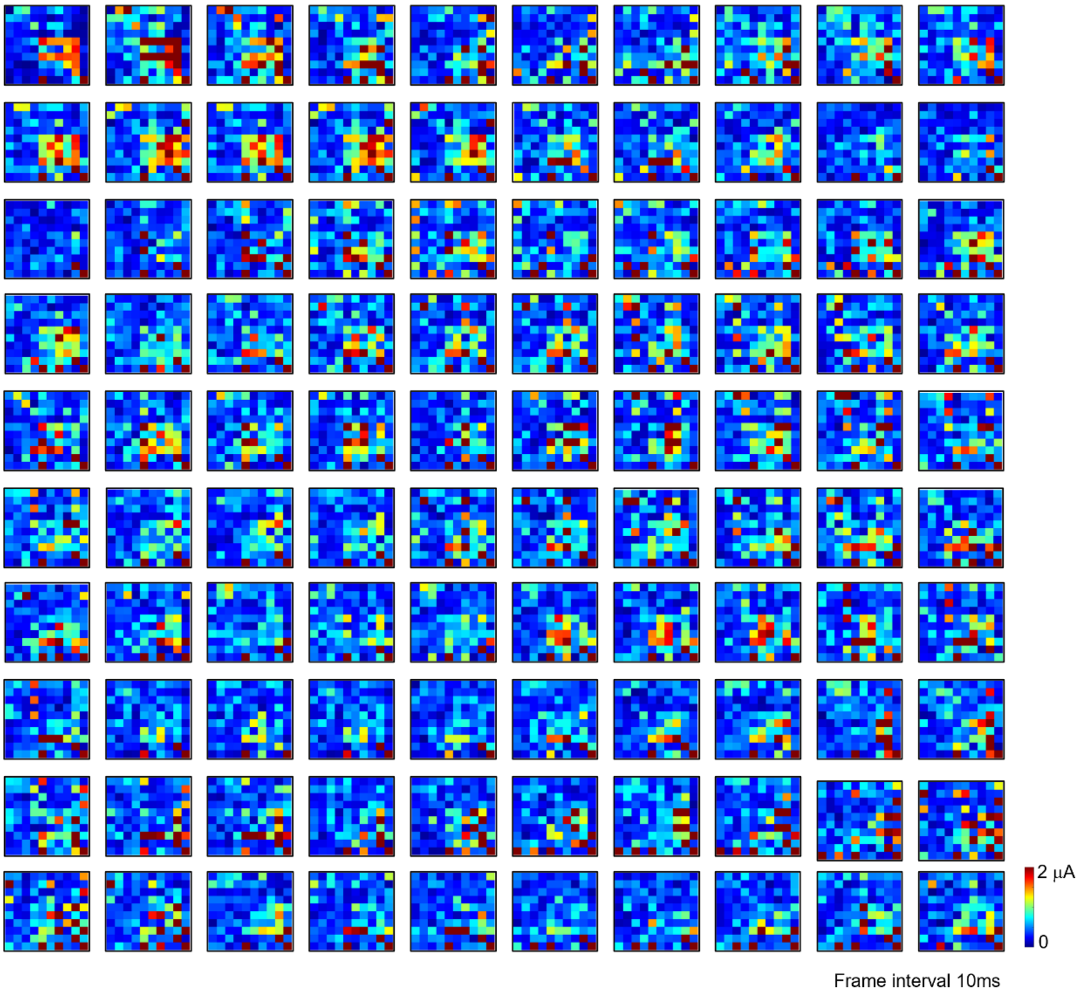

Supplementary Fig. S14. The μ-ECoG mapping result of anaesthetization-induced stimulation on the rat during one second with the frame interval 10ms.

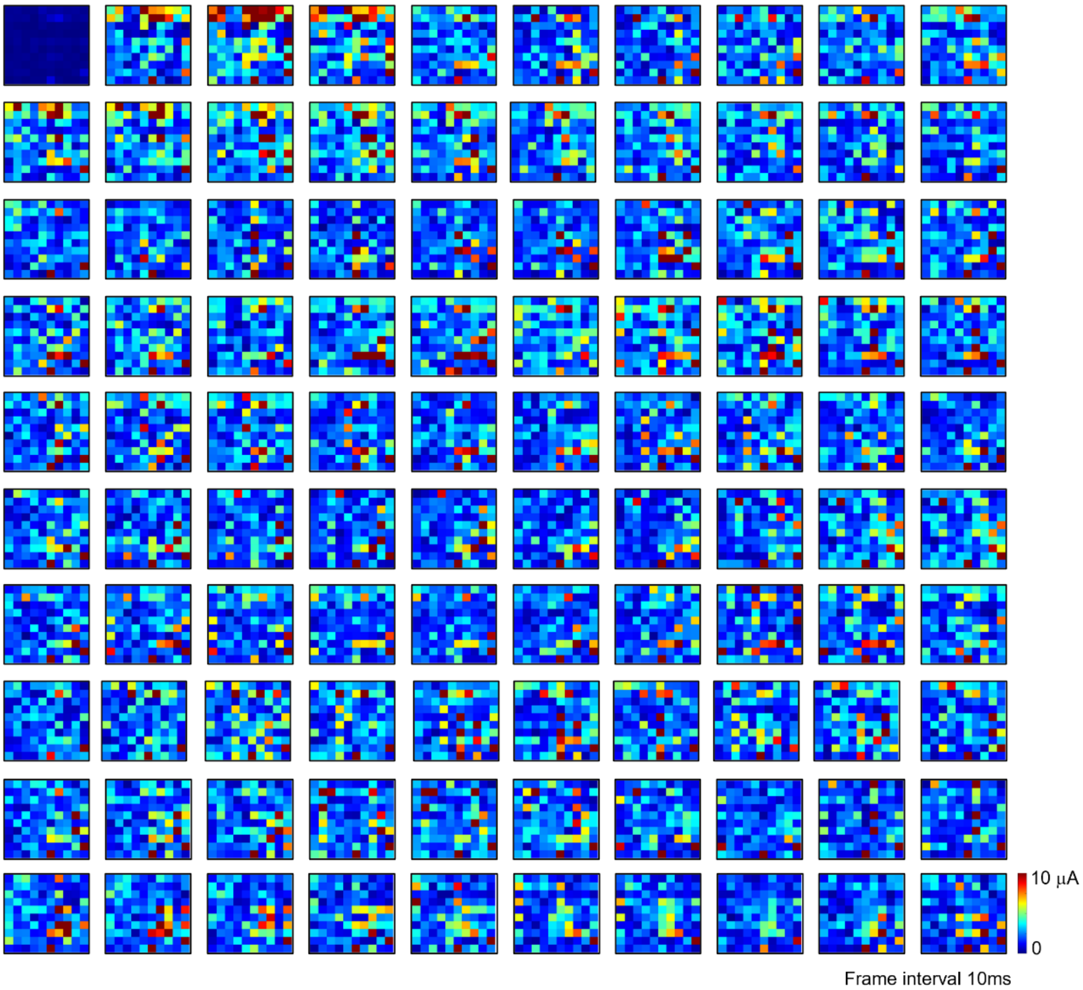

Supplementary Fig. S15 The μ-ECoG mapping result of epilepsy-induced stimulation on the rat during one second with the frame interval 10ms.

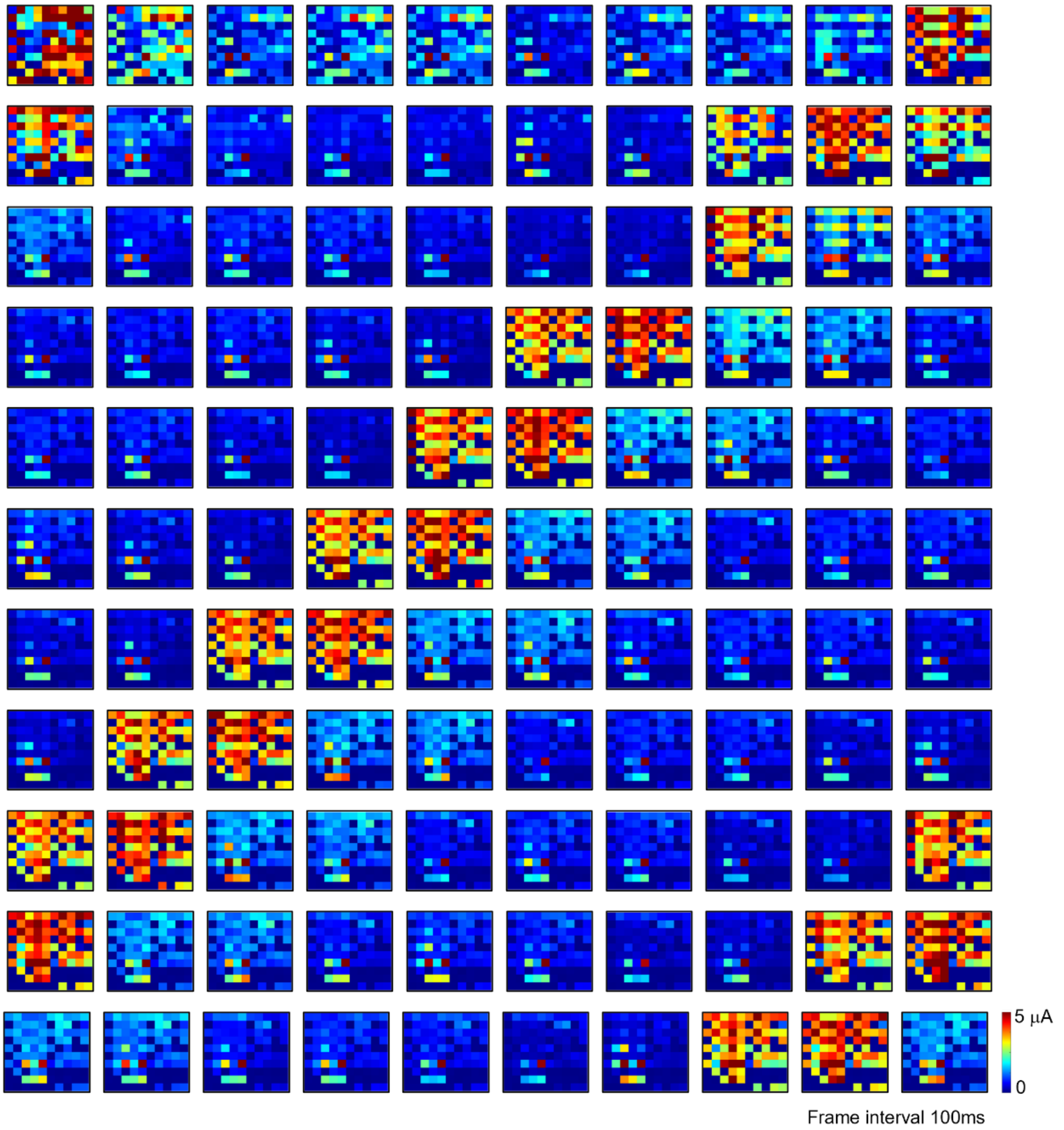

Supplementary Fig. S16. Movie frames show varied spatiotemporal μ-ECoG voltage patterns from all 100 OECT units under electrical-stimuli of 1Hz and 3V on two hind legs, with frame interval of 100ms.

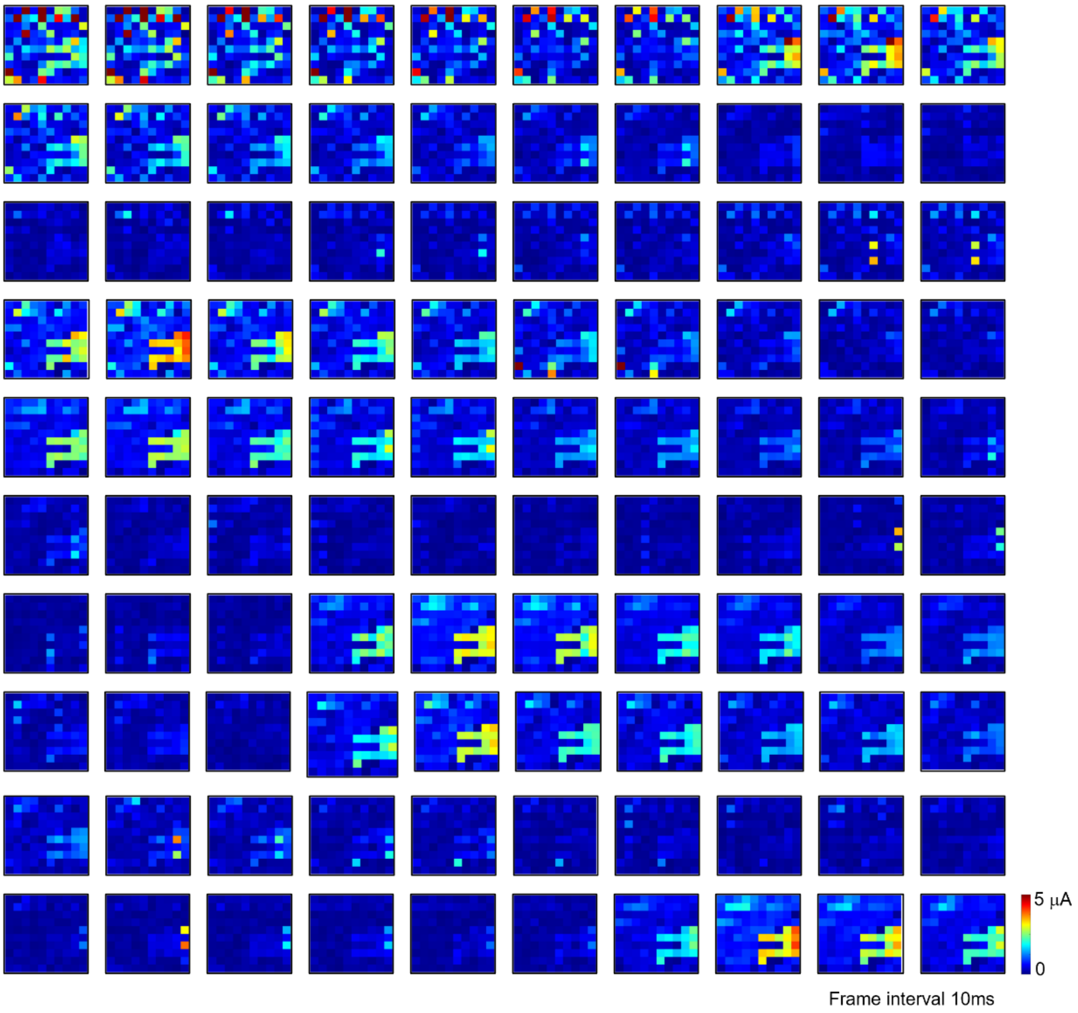

Supplementary Fig. S17. The µ-ECoG mapping result of electrical-induced stimuli of 1Hz, 1V on the rat's left hind leg during one second with the frame interval of 10ms.

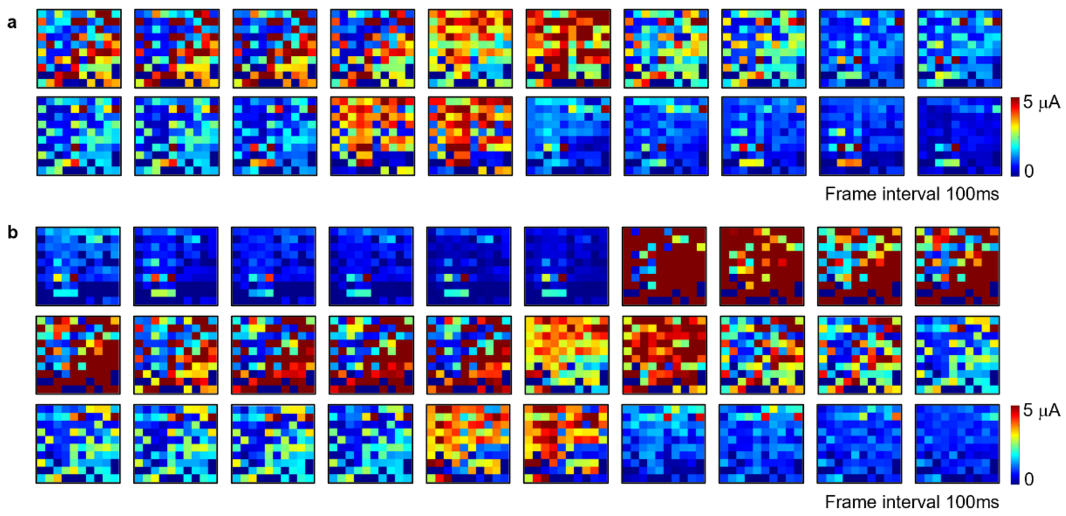

Supplementary Fig. S18. Movie frames show varied spatiotemporal μ-ECoG voltage patterns for endogenous responses evoked by event-related potentials from all 100 OECT units after electrical-stimuli, with frame interval of 100 ms. a. The first endogenous response. **b**, the second endogenous response.

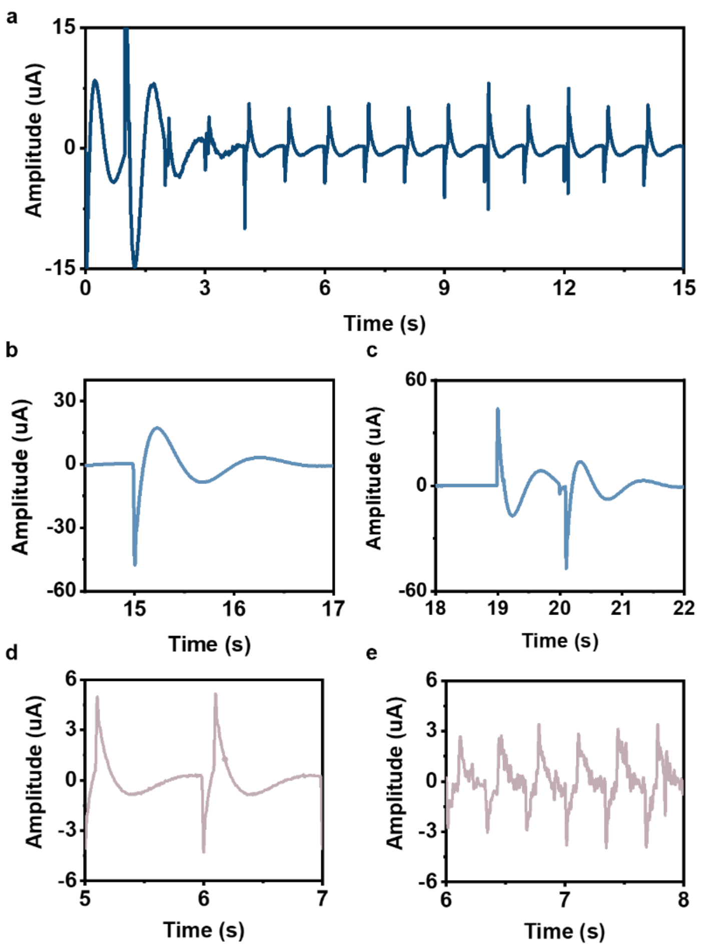

Supplementary Fig. S19. Study of the effects of applying electrical stimulation on μ-ECoG signals on the hind legs of SD rats. a, The exogenous response of SD rats for electrical stimulation with frequency of 1Hz and amplitude of 3V during consecutive 15 seconds, and it can be found that the frequency of μ-ECoG signal generated by somatosensory cortex channels were consistent with that of exogenous stimulation signal. **b**, The first endogenous response evoked by event-related potentials, that appeared within 1 second of cessation of stimulation with a similar waveform but 10-times higher peak amplitude that that of endogenous response. **c**, The second endogenous response evoked by event-related potentials that appeared after 4 seconds after cessation of electrical stimulation, in which the waveform was different from the exogenous response but has a equal peak amplitude with the first endogenous response. **d**, Exogenous response to electrical stimuli with a frequency of 1Hz and amplitude of 3V. **e**, Exogenous response to electrical stimuli with a frequency of 3Hz and amplitude of 1V**.**

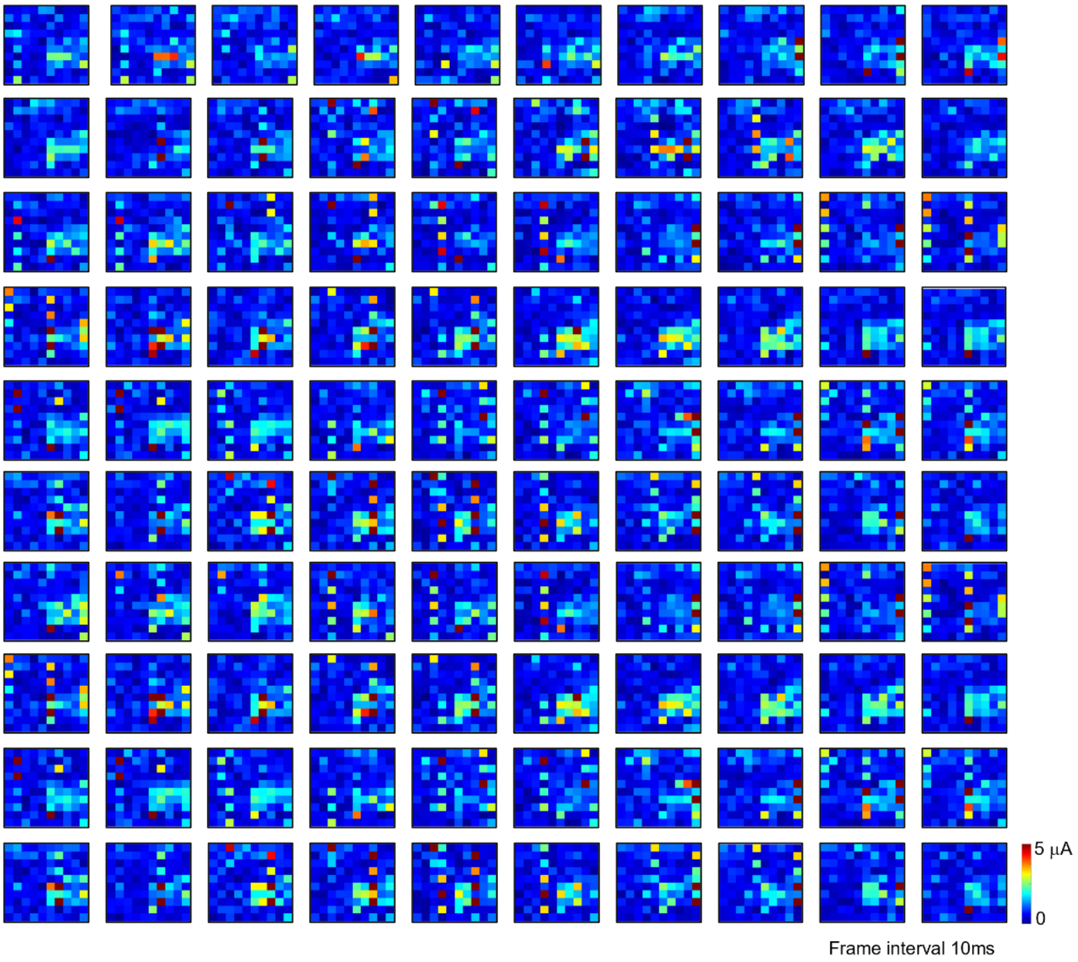

Supplementary Fig. S20. The μ-ECoG mapping result of light-induced stimulation on the rat's right eye during one second with the frame interval of 10ms.

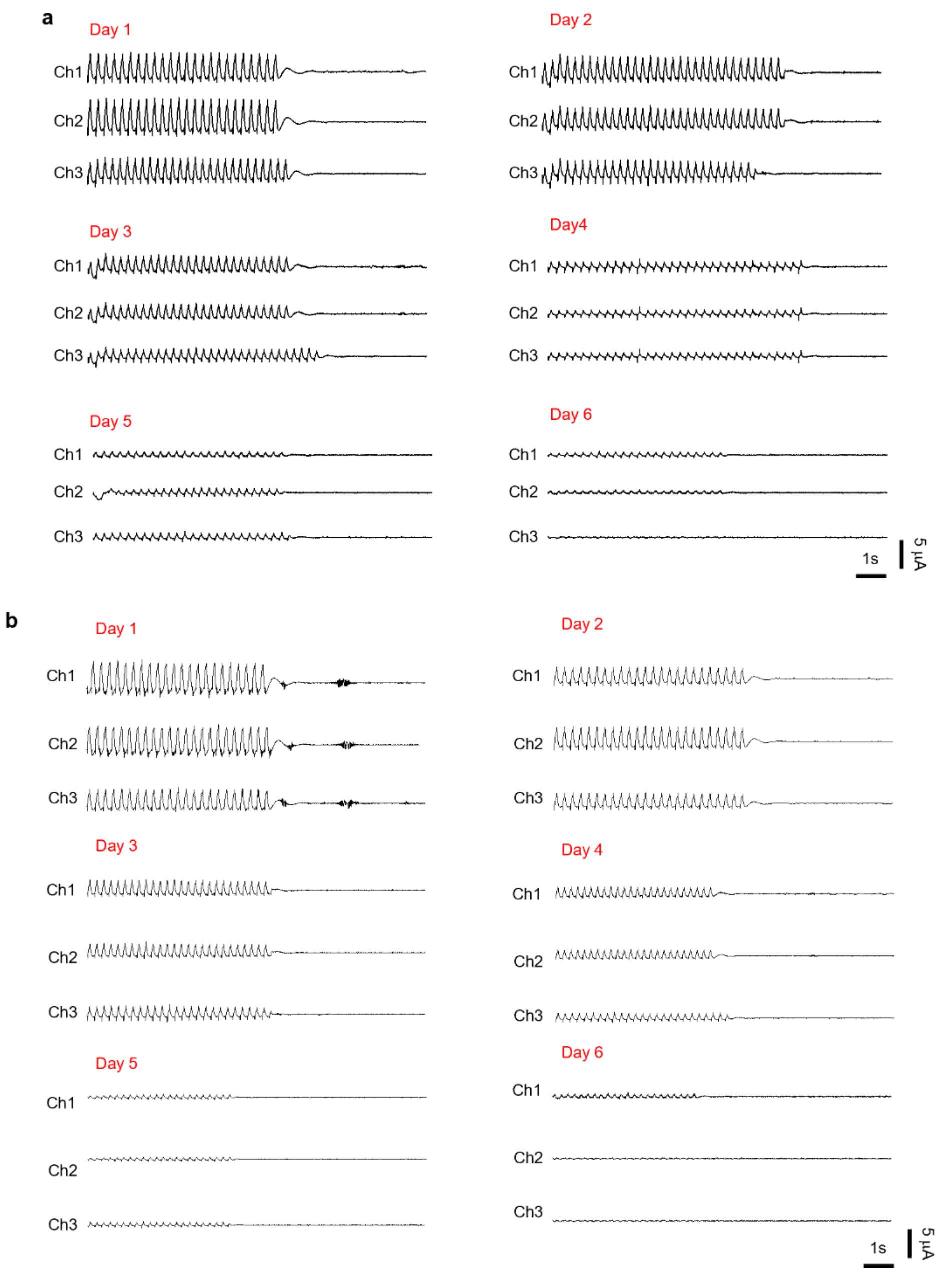

Supplementary Fig. S21. Degradation process of the signal recording capability the transient OECT array *in vivo*, Representative μ-ECoG signals recorded by the transient OECT array on days 1-6, and recordings from three channels in array exhibit large-scale spikes induced by a, electrical stimuli at 3V and 3Hz that consistent with the related local and temporal variations. **b**, electrical stimuli at 3V and 2Hz that consistent with the related local and temporal variations.

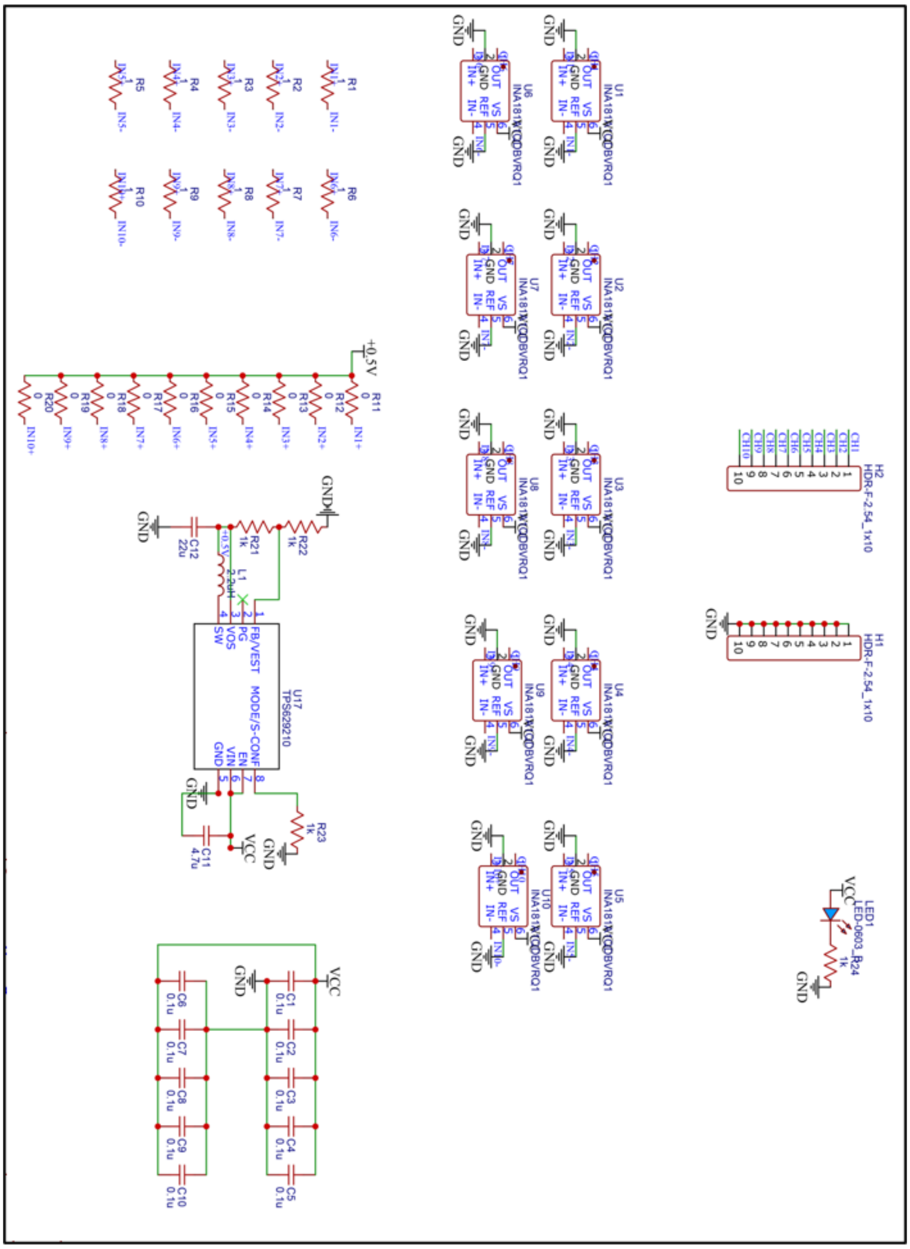

Supplementary Fig. S22. Complete schematics of the data acquisition interface circuit board.

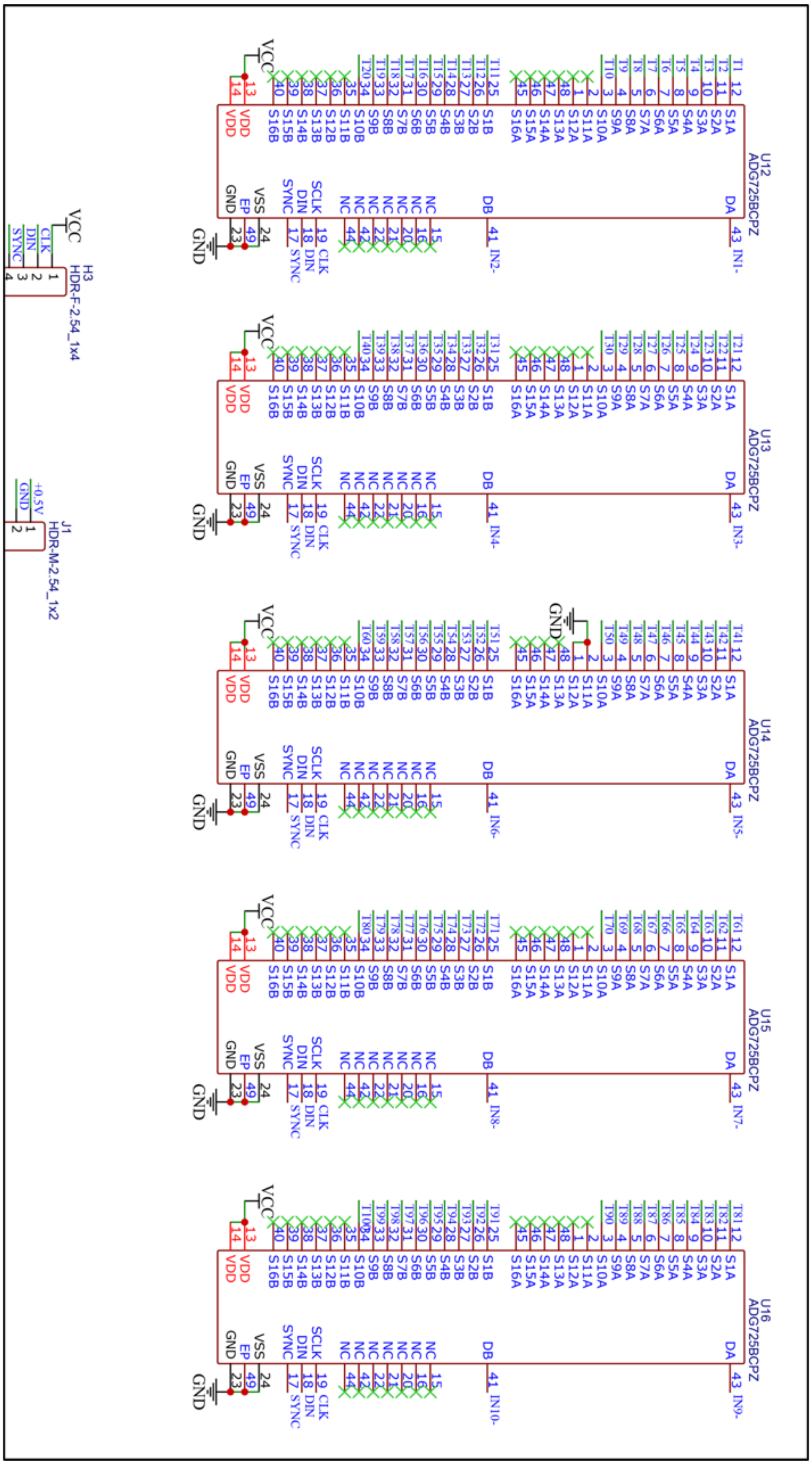

Supplementary Fig. S23. Complete schematics of the data acquisition interface circuit board.

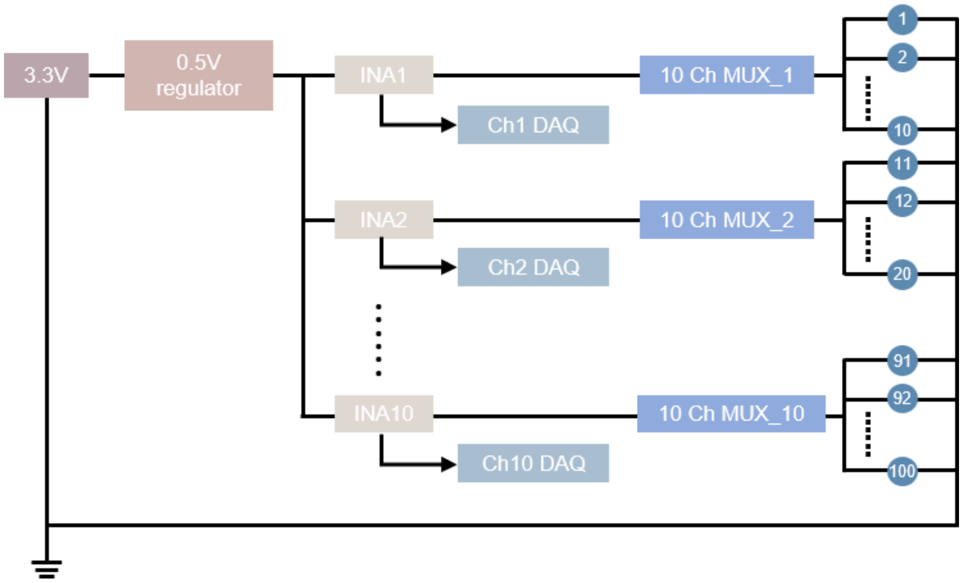

Supplementary Fig. S24. Complete schematics of the data acquisition interface circuit board.

Table S1 Comparison of the key technical information of passive electrode, Si-based FET, and OECT.

	Passive electrode	FET/OTFT	OECT
Signal Amplification	No	Yes	Yes
Transconductance	No	(0.24 mS, 2.0 mS) for graphene-based FET[6-8]<br><br>(4.46 μS, 1.3 mS) for IGZO-based TFT [9-11]<br><br>(9 μS, 120 μS) for carbon nanotubes-based FET[12, 13]<br><br>(5.0 μS, 30 μS) for silicon-based FET [14-16]<br><br>(0.25 μS, 15 μS) for organic TFT [17-20]	(1μS, 100 mS)[2, 3]
Channel Counts	256 [21]	1008 [22]	32 [23]
Signal-to-Noise Ratio	< 20 dB[24]	42 dB[25]	52.7 dB[26]
Lifetime	decades or years[22]	days or weeks[27]	days[28]

Table S2. Summary of the representative reported OECT arrays.

Channel count	Channel size	g_m	Applications	Response time	Biodegradability	Ref.
4 (1 × 4)	40 µm × 10 µm	0.24 mS	monitoring catecholamine neurotransmitters (CA-NTs) in rat brains	several milliseconds	No	[29]
15 (3 × 5)	1mm spacing	1.1 mS	cortical surface	363 µs	No	[30]
4 × 4	spacing of 200 µm, channel size of 30 µm × 5 µm	2.5 mS @vg=-0.1V	action potentials of primary cardiomyocytes	100–200 µs	No	[31]
4 × 4	10 µm × 15 µm	1.1 mS	ECG measurements on the heart surface of rats	60 µs	No	[32]
4 × 4	N/A	12.0 mS	the measurement of cellular action potentials of the cardiac cell line HL-1.	2-5 ms	No	[33]
4 × 4	30 µm × 40 µm	0.5 mS	investigate the behaviour of a newly reported nasopharyngeal cancer cell line NPC43 on epithelial cells	N/A	No	[34]
4 × 4	180 µm × 168 µm	139.0 mS	bioelectrical activity ranging from spreading depolarizations to EMG fasciculations	100 µs	No	[35]
4 × 4	5µm × 5µm	2.0 mS	recording acellular action potential	62 µs	No	[36]
5 × 5	5 mm × 0.2 mm	1.0-2.0 mS	Human hepatoma (HepG2) cancer cells	Less than 1s	No	[37]
4 × 8	40 µm × 30 µm, 40 µm × 20 µm, 30 µm × 20 µm 20 µm × 20 µm.	2.0 mS	Calu-3 cells	100 µs	No	[23]
10 × 10	200 µm × 20 µm	9.0 mS	µ-ECoG signals under narcosis, during epileptic seizure and with electrical stimuli	1.42 mS	Yes	This work

Table S3 Comparison of the performance of state-of-the-art OECTs

Channel	Electrodes (gate/drain-source)	electrolyte	(Wd)/L μm	g_m mS	ref.
PEDOT: PSS	(Ag/AgCl)/Au/Au	0.01 M PBS	0.1	2.3	[38]
PEDOT: PSS	(Ag/AgCl)/Au/Au	0.1 M NaCl	0.46	4.5	[39]
PEDOT: PSS	(Ag/AgCl)/Au/Au	0.1 M NaCl	3.42	12	[40]
PEDOT: PSS	(Ag/AgCl)/Au/Au	0.1 M NaCl	5.4	22	[41]
PEDOT: PSS	(Ag/AgCl)/Au/Au	0.1 M NaCl	28	57	[42]
PEDOT: PSS	(Ag/AgCl)/Au/Au	0.1M KCl	11	12	[43]
PEDOT: PSS	(Ag/AgCl)/Au/Au	Ion gel	50	68.88	[44]
PEDOT: PSTFSI	(Ag/AgCl)/Au/Au	0.1 M NaCl	10	7.6	[45]
PEDOT:PSS/[EMIM][Cl]	(Ag/AgCl)/Au/Au	0.1 M NaCl	1.6	12.9	[46]
PEDOT:PSS/[EMIM][TCM]	(Ag/AgCl)/Au/Au	0.1 M NaCl	20	14.2	[47]
PEDOT:PSS/[EMIM][PF6]/DBSA	Pt/Au/Au	0.1 M NaCl	6	22.7	[48]
PEDOT:PSS/Triton X-100	(Ag/AgCl)/Au/Au	PVA hydrogel	10	54	[49]
PEDOT: PSS	(Ag/AgCl)/Au/Au	0.01 M PBS	1.4	8.9	This work

Table S4. Summary of the characterization of the exogenous components of event-related potentials elicited by different kinds of exogenous stimuli.

Condition	During Narcosis	Epileptic seizure	Electrical stimuli	Light stimuli
Temporal characteristics (ms)	80	60	1s for 1Hz stimuli<br>0.33s for 3Hz stimuli	100
Evoked peak potential (μA)	-0.92, 2.31	-4.99, 2.28	-7.25, 5.45	-1.12, 3.31
Characteristics frequency (Hz)	< 10	60-70	1Hz for 1Hz stimuli<br>3Hz for 3Hz stimuli	50
Response area	all	all	somatosensory	Visual, secondary motor

References:

[1] L. Huang, X. Yang, L. Deng, D. Ying, A. Lu, L. Zhang, A. Yu and B. Duan. *ACS Applied Materials & Interfaces* **2021**, 13.

[2] J. T. Friedlein, R. R. McLeod and J. Rivnay. *Org. Electron.* **2018**, 63.

[3] S. Inal, G. G. Malliaras and J. Rivnay. *Nature communications* **2017**, 8.

[4] Y.-C. Chin, M. Daboczi, C. Henderson, J. Luke and J.-S. Kim. *ACS Energy Letters* **2022**, 7.

[5] C. Duc, G. G. Malliaras, V. Senez and A. Vlandas. *Synthetic Metals* **2018**, 238.

[6] C. Hébert, E. Masvidal-Codina, A. Suarez-Perez, A. B. Calia, G. Piret, R. Garcia-Cortadella, X. Illa, E. Del Corro Garcia, J. M. De la Cruz Sanchez and D. V. Casals. *Advanced Functional Materials* **2018**, 28.

[7] R. Campos, J. Borme, J. R. Guerreiro, G. Machado Jr, M. F. t. Cerqueira, D. Y. Petrovykh and P. Alpuim. *ACS sensors* **2019**, 4.

[8] M. Zhang, C. Liao, C. H. Mak, P. You, C. L. Mak and F. Yan. *Scientific reports* **2015**, 5.

[9] H.-Y. Lee, W.-Y. Ye, Y.-H. Lin, L.-R. Lou and C.-T. Lee. *J. Electron. Mater.* **2014**, 43.

[10] N. Münzenrieder, C. Zysset, L. Petti, T. Kinkeldei, G. A. Salvatore and G. Tröster. *Solid·State Electron.* **2013**, 84.

[11] N. Münzenrieder, P. Voser, L. Petti, C. Zysset, L. Büthe, C. Vogt, G. A. Salvatore and G. Tröster. *IEEE Electron Device Lett.* **2013**, 35.

[12] N. T. Rouf, A. H. Deep, R. B. Hassan, S. A. Khan, M. Hasan and S. M. Mominuzzaman. *Micro & Nano Letters* **2014**, 9.

[13] L. Liu, J. Han, L. Xu, J. Zhou, C. Zhao, S. Ding, H. Shi, M. Xiao, L. Ding and Z. Ma. *Science* **2020**, 368.

[14] M.-Y. Shen, B.-R. Li and Y.-K. Li. *Biosensors and Bioelectronics* **2014**, 60.

[15] R. Tian, S. Regonda, J. Gao, Y. Liu and W. Hu. *Lab on a Chip* **2011**, 11.

[16] B.-R. Li, C.-W. Chen, W.-L. Yang, T.-Y. Lin, C.-Y. Pan and Y.-T. Chen. *Biosensors and Bioelectronics* **2013**, 45.

[17] W. Tang, Y. Fu, Y. Huang, Y. Li, Y. Song, X. Xi, Y. Yu, Y. Su, F. Yan and X. Guo. *npj Flexible Electronics* **2022**, 6.

[18] H. Klauk. *Chemical Society Reviews* **2010**, 39.

[19] C. Wang, W.-Y. Lee, D. Kong, R. Pfattner, G. Schweicher, R. Nakajima, C. Lu, J. Mei, T. H. Lee and H.-C. Wu. *Scientific reports* **2015**, 5.

[20] H. Yoo, S. On, S. B. Lee, K. Cho and J. J. Kim. *Advanced Materials* **2019**, 31.

[21] D. Khodagholy, J. N. Gelinas, T. Thesen, W. Doyle, O. Devinsky, G. G. Malliaras and G. Buzsáki. *Nature neuroscience* **2015**, 18.

[22] C.-H. Chiang, S. M. Won, A. L. Orsborn, K. J. Yu, M. Trumpis, B. Bent, C. Wang, Y. Xue, S. Min and V. Woods. *Science translational medicine* **2020**, 12.

[23] C. Yao, C. Xie, P. Lin, F. Yan, P. Huang and I. M. Hsing. *Advanced Materials* **2013**, 25.

[24] X. Yang, T. Zhou, T. J. Zwang, G. Hong, Y. Zhao, R. D. Viveros, T.-M. Fu, T. Gao and C. M. Lieber. *Nature materials* **2019**, 18.

[25] K. J. Yu, D. Kuzum, S.-W. Hwang, B. H. Kim, H. Juul, N. H. Kim, S. M. Won, K. Chiang, M. Trumpis and A. G. Richardson. *Nature materials* **2016**, 15.

[26] D. Khodagholy, T. Doublet, P. Quilichini, M. Gurfinkel, P. Leleux, A. Ghestem, E. Ismailova, T. Hervé, S. Sanaur and C. Bernard. *Nature communications* **2013**, 4.

[27] S. R. Patel and C. M. Lieber. *Nat. Biotechnol.* **2019**, 37.
[28] H. Lee, S. Lee, W. Lee, T. Yokota, K. Fukuda and T. Someya. *Advanced Functional Materials* **2019**, 29.
[29] K. Xie, N. Wang, X. Lin, Z. Wang, X. Zhao, P. Fang, H. Yue, J. Kim, J. Luo and S. Cui. *Elife* **2020**, 9.
[30] W. Lee, D. Kim, N. Matsuhisa, M. Nagase, M. Sekino, G. G. Malliaras, T. Yokota and T. Someya. *Proceedings of the National Academy of Sciences* **2017**, 114.
[31] X. Gu, C. Yao, Y. Liu and I. M. Hsing. *Advanced Healthcare Materials* **2016**, 5.
[32] W. Lee, S. Kobayashi, M. Nagase, Y. Jimbo, I. Saito, Y. Inoue, T. Yambe, M. Sekino, G. G. Malliaras and T. Yokota. *Science advances* **2018**, 4.
[33] F. Hempel, J. K.-Y. Law, T. C. Nguyen, W. Munief, X. Lu, V. Pachauri, A. Susloparova, X. T. Vu and S. Ingebrandt. *Biosensors and Bioelectronics* **2017**, 93.
[34] S. Y. Yeung, X. Gu, C. M. Tsang, S. W. G. Tsao and I.-m. Hsing. *Sensors and Actuators B: Chemical* **2019**, 297.
[35] J. E. Tyrrell, M. G. Boutelle and A. J. Campbell. *Advanced Functional Materials* **2021**, 31.
[36] Y. Jimbo, D. Sasaki, T. Ohya, S. Lee, W. Lee, F. Arab Hassani, T. Yokota, K. Matsuura, S. Umezu and T. Shimizu. *Proceedings of the National Academy of Sciences* **2021**, 118.
[37] M. Zhang, P. Lin, M. Yang and F. Yan. *Biochimica et Biophysica Acta (BBA)-General Subjects* **2013**, 1830.
[38] A. G. Polyravas, V. F. Curto, N. Schaefer, A. B. Calia, A. Guimera-Brunet, J. A. Garrido and G. G. Malliaras. *Flexible and Printed Electronics* **2019**, 4.
[39] V. Kaphle, S. Liu, A. Al-Shadeedi, C. M. Keum and B. Lüssem. *Advanced Materials* **2016**, 28.
[40] M. Z. Szymański, D. Tu and R. Forchheimer. *IEEE Trans. Electron Devices* **2017**, 64.
[41] J. Rivnay, P. Leleux, M. Ferro, M. Sessolo, A. Williamson, D. A. Koutsouras, D. Khodagholy, M. Ramuz, X. Strakosas and R. M. Owens. *Science advances* **2015**, 1.
[42] M. J. Donahue, A. Williamson, X. Strakosas, J. T. Friedlein, R. R. McLeod, H. Gleskova and G. G. Malliaras. *Advanced Materials* **2018**, 30.
[43] J. Rivnay, S. Inal, B. A. Collins, M. Sessolo, E. Stavrinidou, X. Strakosas, C. Tassone, D. M. Delongchamp and G. G. Malliaras. *Nature communications* **2016**, 7.
[44] Y. Yan, Q. Chen, X. Wu, X. Wang, E. Li, Y. Ke, Y. Liu, H. Chen and T. Guo. *ACS Applied Materials & Interfaces* **2020**, 12.
[45] S. Inal, J. Rivnay, A. I. Hofmann, I. Uguz, M. Mumtaz, D. Katsigiannopoulos, C. Brochon, E. Cloutet, G. Hadziioannou and G. G. Malliaras. *J. Polym. Sci., Part B: Polym. Phys.* **2016**, 54.
[46] X. Wu, A. Surendran, M. Moser, S. Chen, B. T. Muhammad, I. P. Maria, I. McCulloch and W. L. Leong. *ACS applied materials & interfaces* **2020**, 12.
[47] X. Wu, A. Surendran, J. Ko, O. Filonik, E. M. Herzig, P. Müller-Buschbaum and W. L. Leong. *Advanced Materials* **2019**, 31.
[48] L. Wang, Q. Sun, L. Zhang, J. Wang, G. Ren, L. Yu, K. Wang, Y. Zhu, G. Lu and H. D. Yu. *Macromol. Rapid Commun.* **2022**, 43.
[49] J. Ko, X. Wu, A. Surendran, B. T. Muhammad and W. L. Leong. *ACS applied materials & interfaces* **2020**, 12.
